# *Virgulinella fragilis* in the North Adriatic Coastal Sediments: A New Non-Indigenous Benthic Foraminiferal Taxon?

**DOI:** 10.3390/biology14040421

**Published:** 2025-04-14

**Authors:** Roberta D’Onofrio, Maria Letizia Vitelletti, Francesco Riminucci, Veronica Rossi, Lucilla Capotondi

**Affiliations:** 1 Institute of Marine Sciences (ISMAR), National Research Council (CNR), Arsenale, Tesa 104, Castello 2737/f, 30122 Venezia, Italy; marialetizia.vitelletti@cnr.it; 2 Institute of Marine Sciences (ISMAR), National Research Council (CNR), Via Piero Gobetti, 101, 40129 Bologna, Italy; francesco.riminucci@bo.ismar.cnr.it (F.R.); lucilla.capotondi@bo.ismar.cnr.it (L.C.); 3 PROAMBIENTE Consortium, Tecnopole Bologna CNR, Via Piero Gobetti, 101, 40129 Bologna, Italy; 4 Dipartimento di Scienze Biologiche, Geologiche e Ambientali, Università di Bologna, Via Zamboni 67, 40126 Bologna, Italy; veronica.rossi4@unibo.it

**Keywords:** benthic foraminifera, non-indigenous species, North Adriatic Sea, LTER, biomonitoring, habitat suitability

## Abstract

The Mediterranean Sea is considered a hotspot for bioinvaders. Nonetheless, information on non-indigenous benthic foraminifera is still fragmented. This study documents for the first time the presence along the northwestern Adriatic coast of the non-indigenous benthic foraminifera species *Virgulinella fragilis*, Grindell and Collen (1976). Due to the low abundance recorded in the study area, the presence of this species may represent an early colonization phase. We discuss the temporal and spatial patterns of *V. fragilis* arrival in the Mediterranean and Adriatic Seas, and we hypothesize stowaway transport (via ship fouling or ballast water) as the main introduction pathway. Morphological test analyses suggest that *V. fragilis* prefers a low oxygen content, consistent with the ecological requirements reported for this taxon in the literature. The application of Maximum Entropy (MaxEnt) modeling indicates that the key factor influencing the presence of *V. fragilis* in the Mediterranean basin is the bacterial concentration expressed as NO_3_. Projections under future climate scenarios (RCP 4.5) point to a decline of habitat suitability conditions, making widespread invasion unlikely in the Mediterranean. We emphasize the importance of continuous biomonitoring for early detection of alien species, improving our understanding of invasion dynamics and enabling prompt conservation actions, especially in regions impacted by anthropogenic activities.

## Simple Summary

This study documents the presence of a non-native benthic foraminifera, *Virgulinella fragilis*, along the northwestern Adriatic coast, marking its first recorded presence in the area. This species, which lives in **marine** sediments, could spread and impact local ecosystems. We suggest that human activities, such as marine shipping, may have contributed to its arrival, though the exact transport method remains unclear. The test morphology of *Virgulinella fragilis* suggests that this species prefers low-oxygen environments. Using machine learning methods, we modeled its potential spread in the Mediterranean, finding that, besides oxygen depletion, riverine nutrient input, particularly bacterial nitrates, might play a key role in its distribution. However, climate change may reduce its potential suitable habitats, making widespread invasion unlikely in the Mediterranean. **The results** also emphasize the importance of continuous monitoring of marine species, especially in regions impacted by human activities, to enable early detection of non-native species and prompt action to protect ecosystems.

## 1. Introduction

Anthropogenic pressures on marine ecosystems have significantly increased in recent decades, with future projections indicating further escalation [1,2,3,4,5], leading to profound and potentially cascading consequences for human societies [1,5,6,7,8,9,10,11]. Along with land-use changes, exploitation, climate change, and pollution, bioinvasions stand as direct anthropogenic drivers of biodiversity loss, contributing to 60% of global extinctions [12,13].

Invasive species not only threaten biodiversity but also pose risks to the economy, food security, and human health [12]. Given their global impact on ecosystems [14,15,16], urgent conservation actions are needed [12]. As a result, international regulations have been implemented to address this issue through measures such as identification, prioritization, control, prevention, and eradication (e.g., [17,18,19,20,21,22,23,24,25]).

In the context of biodiversity conservation, organizations (i.e., International Union for Conservation of Nature, IUCN; Intergovernmental Science-Policy Platform on Biodiversity and Ecosystem Services, IPBES; etc.) and regulatory frameworks developed a glossary that marks clear distinctions between the diverse types of introduced species. Non-indigenous species (NIS), also referred to as exotic, alien, or non-native, are defined as organisms introduced outside their natural range due to human actions, whether intentionally or accidentally [26]. Invasive alien species (IAS) are a subset of established NIS that spread or have the potential to spread, causing significant negative impacts on biodiversity, ecosystems, economies, or human health [26]. Therefore, though all IAS are NIS, not all NIS are invasive. Natural climate shifts or transport through natural means can also result in secondary introductions. However, these changes in species distribution, if occurring without human involvement, do not classify a species as a NIS but rather as an introduced species (IS), or introduced individual (II) at the initial phase of the spreading process [27].

In marine and coastal environments, NIS and IAS are often introduced through maritime transport, aquaculture, aquarium trade, and artificial waterways such as the Suez Canal (e.g., [28,29,30]). One of the most prominent regions affected by IAS is the Mediterranean Sea, which has become a hotspot for bioinvaders, mainly due to the opening of the Suez Canal, shifting climate trends, and increased maritime traffic (e.g., [31,32]). The eastern basin of the Mediterranean is notably impacted by Lessepsian species (i.e., Red Sea origin) of multiple taxonomic groups while other Mediterranean ecoregions are more affected by invasive macrophytes [28,31,32,33,34,35,36,37,38,39]. Consequently, bio-pollution [40] due to IAS has become a central issue in Mediterranean countries, which have enacted protocols addressing the problem in line with global and European initiatives [41,42,43].

Efforts to manage NIS and IAS are supported by information systems such as the European Alien Species Information Network (EASIN) [44,45], Aquatic Non-Indigenous and Cryptogenic Species (AquaNIS), and the World Register of Introduced Marine Species (WriMS) [46]. These databases are invaluable tools for risk management in line with EU and regional regulations. However, challenges remain in obtaining detailed ecological data, particularly in vulnerable regions like the Mediterranean and Black Sea [47], and for smaller taxa, such as foraminifera, for which records are often scarce [48].

Foraminifera, single-celled eukaryotes with mineralized shells, play a key role in marine ecosystems due to their high diversity, short life cycles, and sensitivity to environmental changes, which makes them relevant indicators of both natural and anthropogenic stressors [49,50,51,52,53,54,55]. Additionally, their well-preserved shells in fossils and recent sediments make them valuable tools for studying past and present biotic and environmental shifts [56,57,58].

In particular, benthic foraminifera (BF) are crucial for marine ecosystem functions, contributing significantly (~50%) to the sediment biomass in shallow seabeds [59,60], as important food sources for larger marine organisms [61,62,63] and due to their key role in biogeochemical cycles, aiding the remineralization of carbon, nitrogen, and other nutrients [64,65,66,67,68].

Despite their ecological relevance, data on BF NIS and IAS remain fragmented and potentially underestimated. While larger marine organisms like fish and bivalves are well-documented due to fishing and shell collection [69,70], smaller taxa like BF are often overlooked until their populations reach significant densities [71,72]. This gap is due to the inadequate monitoring of small taxa and the limited involvement of non-specialists or citizen scientists in monitoring microorganisms, as their microscopic nature requires specialized expertise and equipment, restricting observations mainly to researchers [73].

Nonetheless, invasive BF species, such as *Amphistegina lobifera* [74] and *Trochammina hadai* [75], pose profound concerns as their spread in various regions has been linked to alterations in both biodiversity and community structures, thus threating ecosystem functions reliant on the balance of these microorganisms [76,77,78,79,80,81]. To mitigate these impacts, continuous research and monitoring are essential. Regular surveys, public participation, and re-evaluation of historical research collections can help improve the understanding of IAS distribution and their effects on ecosystems (e.g., [82,83]).

In this study, we testify for the first time to the presence of *Virgulinella fragilis* [84], a new potential non-indigenous foraminiferal species (foram-NIS), in the Adriatic Sea. This species was identified in the North Adriatic Foraminifera Collection (NAdFC), obtained through biomonitoring activities conducted at the North Adriatic Long-Term Ecological Research (LTER) site “Delta del Po e Costa Romagnola” [85,86]. Additionally, we formulate hypotheses concerning its presence in the area and the potential introduction dynamics, while also providing perspectives on suitable spreading areas in the Mediterranean Sea and future trends. Our finding highlights the importance of long-term biomonitoring to ensure early detection of NIS/IAS, particularly in regions strongly impacted by anthropogenic activities, climate shifts, and environmental changes, such as the North Adriatic [87,88].

## 2. *Virgulinella fragilis*: Potentiality for Invasiveness

In terms of ecological preferences, *V. fragilis* represents a highly specialized BF, tolerant to nearly anaerobic conditions and adapted to sulfide-enriched habitat, which exemplifies the complex interactions between microorganisms and their environments [89,90,91,92,93]. Tsuchiya et al. [93] explored the cytological and genetic characteristics of endobiotic bacteria and kleptoplasts associated with *V. fragilis*, revealing that this foraminifer possesses specific δ-proteobacteria at its cell periphery and maintains a relatively stable bacterial community compared to other foraminifera like *Stainforthia* spp. This indicates a specialized intracellular bacterial flora that may significantly contribute to the organism’s metabolic processes. Endosymbionts may be both sulfide oxidizers and sulfate reducers [89,93]. Furthermore, kleptoplasts from various diatom species were identified within *V. fragilis*, suggesting a complex symbiotic relationship that enhances its survival in nutrient-poor environments. This species showcases unique adaptations that allow it to flourish in extreme conditions such as oxygen-depleted and sulfide-rich habitats, thereby highlighting its potential for invasiveness in regions where these conditions prevail.

Additionally, a significant study conducted by Tsuchiya et al. [94] investigated the genetic and morphological divergence of *V. fragilis* across global populations, revealing extremely low genetic divergence among specimens and indicating a high level of genetic stability despite the species’ disjunct distribution across marine environments. This low degree of genetic divergence across the global population suggests a successful adaptation strategy that allows *V. fragilis* to thrive in diverse yet challenging environments, thus likely confirming the potential for an invasive behavior. Interestingly, a similar pattern highlighting minimal molecular genetic differentiation between populations was also observed for the highly invasive taxon *T. hadai* [78].

## 3. Study Area

### 3.1. The Northern Adriatic Sea

The Northern Adriatic Sea (NAS) is a key sector of the epicontinental Adriatic basin, known for the complex interaction between freshwater, sediments, and currents, which have significant implications for the regional climate, marine ecosystems, and sediment dynamics (e.g., [95,96,97]). It occupies the flooded seaward extension of the Po Plain, forming the largest continental shelf in the Mediterranean, distinguished by a gentle axial topographic gradient (e.g., [95,98,99,100]).

The NAS features a cyclonic circulation with a northwest inflow along the eastern Croatian coast (Eastern Adriatic Coastal Current—EACC), a southeast outflow along the western Italian coast (Western Adriatic Coastal Current—WACC), and a local northern gyre near the Po River mouth (NAdG—North Adriatic Gyre) (e.g., [96,101]). This general pattern is greatly influenced by winds, mainly Bora and Scirocco, thermal conditions, and lateral freshwater inputs (e.g., [95,102,103,104]).

About one-third of the Mediterranean’s freshwater inflow, averaging over 2400 m^3^/s, is supplied into the NAS primarily from northern and western Adriatic rivers [105,106,107,108,109,110]. The Po River alone contributes ~58% of this input [109], following a mixed Alpine/Apennine regime with seasonal peaks in late autumn and spring, due to precipitation and snowmelt, and a minimum in mid-summer [111]. In the past decade, freshwater input has decreased due to extreme droughts [109,112]. Climate models predict altered Po discharge patterns, with reduced flow from May to November, contributing to further warming in the NAS [113,114].

The seabed of the NAS primarily consists of sandy–muddy sediments, with major contributions from rivers along the northern and western Adriatic coasts. The Po River serves as the main sediment source, delivering approximately 12.2 Tg/year, followed by the Eastern Alpine (3.2 Tg/year) and the Apennine rivers (16.9 Tg/year) [108,115,116]. In this region, river input exceeds sediment accumulation, resulting in a counter-clockwise sediment transport pattern driven by the WACC towards the central and southern Adriatic areas, which affects sediment distribution, with sands nearshore and mud further offshore [117,118,119]. Within the western NAS, the “Adriatic mud-belt” (at depths of 20–40 m) from the Po outlets to Ravenna shows high accumulation rates, particularly south of the Po Delta, where oxygen deficiency occurs due to organic matter degradation [120,121]. The highest mass accumulation rates are observed near the Po outlets, especially following floods [88,99]. Accumulation rates in the prodelta range from 1 to 5 cm/year, peaking near the Pila and Goro tributaries [116,122]. These sediments are rich in refractory organic matter from terrestrial sources, with elevated nickel and chromium levels linked to ultramafic rock formations in the river’s catchment [123,124,125].

In terms of anthropic pressure, the Po Valley, with its dense population and intense exploitation, exerts a significant impact on the NAS through agricultural runoff, industrial discharge, and urban wastewater, contributing to nutrient loading and pollution [112,125,126,127]. This increased nutrient input, including nitrates and phosphates, leads to eutrophication, algal blooms, and oxygen depletion in bottom waters, especially in the western NAS inner shelf [112,121,128]. Coastal urbanization and land reclamation have altered habitats and exacerbated coastal erosion [127,129]. Additionally, major ports like Venice, Trieste, Ravenna, and Ancona impact the marine environment through shipping, dredging, and industrial activities, introducing NIS and disturbing sediments [128,130,131]. Fishing and aquaculture also contribute to benthic ecosystem disturbance causing habitat degradation, and pollution from nutrient-rich fish farm waste [128,132,133].

### 3.2. Sampling Stations at the LTER “Delta del Po e Costa Romagnola” Site

**Figure 1:**
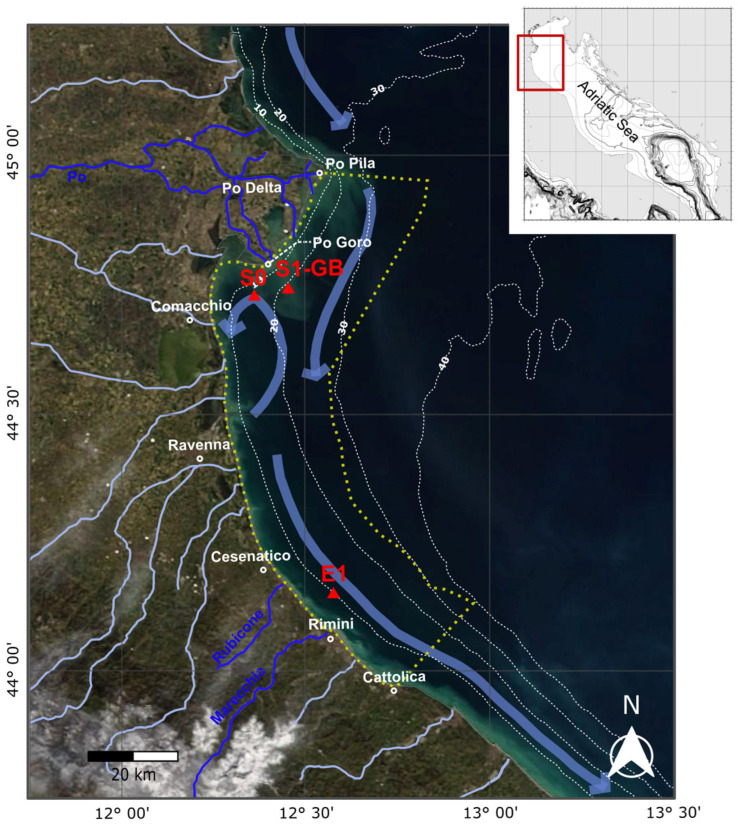
Satellite image showing location of the LTER DPCR area (yellow dashed lines) and the S0, S1-GB and E1 sampling stations (red triangles) within the western sector of the NAS. Isobaths (white lines) every 10 m are from [151]. Blue arrows indicate a simplified pattern of sediment flux, influenced by the NAS surface circulation after [149]. Hydrographic network of the main rivers contributing to sediment input at these sampling stations (Po, Rubicone, and Marecchia) is shown in blue and is sourced from [125].

This study focuses on the Italian LTER Site “Delta del Po e Costa Romagnola” (DPCR hereafter, https://deims.org/6869436a-80f4-4c6d-954b-a730b348d7ce, accessed on the 7 January 2025), located in the western sector of the NAS (Figure 1) and extending from the mouth of the Pila Po to the offshore of Cattolica (from 44.97° N to 43.97° N). The LTER network aims at understanding ecosystem dynamics through interdisciplinary research with the goal to address complex ecological questions and contribute to European environmental monitoring [86].

Representing a hub for multidisciplinary research within the NAS, the DPCR has been extensively studied. The topics covered so far include the physical interactions between the Po River and the Adriatic Sea circulation [134,135,136,137], the sediments and heavy metal distribution [88,116,125,138,139,140,141,142,143,144], and, more recently, the BF composition [85,145].

The LTER DPCR site has been the subject of a systematic study on BF communities since 2016 [85,144], conducted through seasonal sampling of seabed sediments. The findings outlined in this study have emerged from continuous monitoring activities at the DPCR site.

The three DPCR sampling stations considered in our investigation are located slightly south of the Po Delta, S0 and S1-GB, and at the Rimini offshore, E1 (Figure 1), at depths of 13.5 m, 22.5 m, and 10.5 m, respectively. At these stations, two autonomous platforms, the S1-GB beacon and E1 buoy, nearly continuously measure physico-chemical oceanographic parameters [146,147]. This provides a long-term series of environmental data, enabling more reliable interpretations than those derived from sporadic measurements. The S0, S1-GB, and E1 areas are influenced by distinct near-bottom hydrodynamic conditions, with S0 and S1-GB being less affected by the WACC bottom currents than E1 [85,148,149,150]. Bottom sediments at the S1-GB and S0 stations are dominated by clay and silt from the Po River [85,88,125]. In contrast, E1 sediments are more heterogeneous, consisting of silty clays with high sand percentages, sourced primarily from the northern Apennines rivers, with some contribution from the Po River [125].

## 4. Materials and Methods

### 4.1. Foraminiferal Samples and Preparation

The BF samples used in this study are part of the NAdFC, a multi-year biomonitoring time series that has been collected since 2016 and is hosted at the National Research Council-Institute of Marine Science (CNR-ISMAR) in Bologna, Italy. The NAdFC consists of various micropaleontological slides containing BF specimens from sediment samples collected at the seabed of the S1-GB (Po Delta) and E1 (Rimini offshore) LTER-DPCR stations (Appendix A, Table A1). From 2016 to the present, a total of 23 and 15 BF samples have been collected at the S1-GB and E1 stations, respectively, while another 8 BF samples were derived from depth transects (depth ranging between 10.5 m in S0 and 30 m in S3) collected during research cruises and centered on S1-GB (Appendix A, Table A1).

The NAdFC sediment samples were collected using a box corer on board the research vessels *Dallaporta* and *Minerva Uno* during expeditions in April and December 2016, March 2017, and February 2019 [152,153,154], or employing a Van Veen grab during ordinary maintenance activities of the S1-GB and E1 autonomous meteo-oceanographic systems. Each box core and grab was subsampled on-site, and approximately 30 mL of sediment from the 0–1 cm interval was collected for BF analysis. The samples were treated following the FOBIMO protocol [51], involving storage in buffered ethanol (>70%) stained with rose Bengal (2 g/L) for at least 14 days to differentiate between living and dead foraminiferal tests. Subsequently, the samples were gently washed and dried at 50 °C in the ISMAR-CNR laboratory in Bologna.

BF specimens were picked from the sediment fraction >63 μm of each sample, identified and counted under an optical stereomicroscope (model Zeiss SteREO Discovery V.8, Oberkochen, Germany), and then mounted on cardboard microslides stored at CNR-ISMAR in Bologna (Italy). The >63 μm size fraction was selected to obtain information on small-sized species (e.g., [155,156]). Identification at the genus level was performed according to the most used taxonomical study on foraminiferal genera [157], while species were determined according to some important studies in the Mediterranean area (e.g., [85,158,159,160,161]) and to the World Modern Foraminifera Database [162].

NAdFC BF specimens are currently undergoing cataloguing, taxonomic revision, digitization, and dataset harmonization in the frame of the DiSSCo (Distributed System of Scientific Collections) Infrastructure and the ITINERIS (Italian Integrated Environmental Research Infrastructure System) Project activities. These initiatives aim to share data and metadata through open institutional archives, the ITINERIS-HUB and the Global Biodiversity Information Facility (GBIF) portal, following FAIR (findable, accessible, interoperable, and reusable) principles. A subset of NAdFC taxa occurrences was recently published on GBIF [145].

### 4.2. Morphological Analyses

*Virgulinella fragilis* tests were photographed using an optical stereomicroscope (model Zeiss SteREO Discovery V.8). Optical images were acquired with a 16× Ocular, S1.0x Objective Plan and processed via the microscope imaging software Olympus EPview (v. 2023, 64 bit). Additional images and a chemical–mineralogical characterization of *V. fragilis* tests were conducted using elemental chemical mapping and point analyses with an Environmental Scanning Electron Microscope (ESEM) Zeiss EVO LS 10, equipped with an Energy-Dispersive Spectroscopy (EDS) device from Bruker (Billerica, MA, USA), featuring the Quantax system and Esprit software (v. 1.9, 64 bit) at the Institute of Microelectronics and Microsystems (CNR-IMM). The ESEM allowed operation in low vacuum mode (approximately 0.3 Torr in this study), which helped reduce charging effects caused by the electron beam on non-conductive samples. *Virgulinella fragilis* specimens were mounted on stubs and imaged using back-scattered electrons at 20 kV to visualize their test morphologies. As test porosity may vary between older and younger chambers, the porosity, i.e., distribution, size, and shape of *V. fragilis* pores, was investigated only on the penultimate chamber, through qualitative and quantitative approaches, to understand the relation between test morphologies and environmental characteristics. Specifically, we qualitatively assessed the pore density (PD) and pore shape (PSh), while quantitatively, we measured the pore size (PS). Pores were digitally photographed, and they were measured for their maximum length and maximum width. These characteristics and measurements were then compared with those reported by Tsuchiya et al. [94].

### 4.3. Habitat Suitability Modeling

To understand the potential spread of non-indigenous species, a powerful tool implemented in ecology are the Habitat Suitability Models (hereafter HSMs). These models aim to reproduce the potential distribution of species by correlating their presence with the environmental patterns characterizing the area of interest.

In this study, we implemented the Maximum Entropy (hereafter MaxEnt) machine learning method, an algorithm that evaluates the density present across a landscape of environmental factors to return a species probability distribution [163,164,165].

HSMs are often populated with occurrence data (presence-only) and a set of environmental factors considered relevant to the species presence. The presence data, consisting of latitude and longitude coordinates, were retrieved during the sampling campaigns conducted at the NAS DPCR stations. Additional points of *V. fragilis* occurrence were obtained by Delliou et al. [166] in the Thermaikos Gulf (Greece). This allowed us to obtain a dataset consisting of 6 occurrences in total. The environmental factors identified as relevant for the presence of *V. fragilis* and employed in the suitability analysis are the bacterial concentration expressed as nitrogen, diatom concentration expressed as carbon, dissolved molecular oxygen concentration in seawater, nitrate concentration in seawater, and semi-refractory dissolved organic matter concentration expressed as carbon, all considered as near-bottom quantities. The selected features were retrieved and sampled from the “Centro Euro-Mediterraneo sui Cambiamenti Climatici Data Delivery Sistem” (hereafter CMCC-DDS), a datacenter containing the Mediterranean Sea marine biogeochemistry simulation under CMIP5 future scenario projections for the 21st century. This dataset provides a set of biogeochemical parameters over the Mediterranean Sea and describes the evolution under the future RCP 4.5 scenario. For study purposes, we correlated the occurrence point with two time scenarios—the first is a historical period in the recent past (2013–2023); the second is a future RCP 4.5 climate change scenario (2050–2099)—to investigate the potential shift of the species distribution. All the variables were sampled on the same grid with 0.04° resolution, bracketing to the geographical domain of the Mediterranean Sea, interpolated using the “nearest neighbor” method.

Before performing the suitability analysis, we conducted a preliminary investigation to identify the optimal parameters for tuning in order to achieve the best goodness of fit and accurately model species distribution. Additionally, the dataset was divided into training (70%) and testing (30%) subsets. The training dataset was used to learn the relationships between environmental variables and species occurrences, while the testing dataset served to evaluate the model’s performance. The goodness-of-fit was assessed using the Area Under the Curve (AUC) metric, which is essential for determining the model’s ability to distinguish between true positive and false positive instances. AUC values close to 1 indicate a strong distinction between the two classes, whereas values near 0 suggest an inability to correctly differentiate them. Finally, a suitability map was generated to represent the probability of finding suitable habitats for the species. The output map ranged from 0 to 1, where 0 corresponds to the least favorable conditions and 1 represents the most suitable conditions for the species.

When applying machine learning approaches such as MaxEnt, it is crucial to consider potential overfitting, where the model adapts itself to the parameters rather than identify true ecological relationships, as well as underfitting, where the model lacks the flexibility to accurately describe species–environment interactions. To address these concerns, during the model setup phase, we carefully adjusted the regularization parameter—an inbuilt MaxEnt function specifically designed to mitigate overfitting—and we decided to keep only the linear feature class.

## 5. Results

### 5.1. Occurrence of Virgulinella fragilis at the NAS DPCR Stations

**Figure 2:**
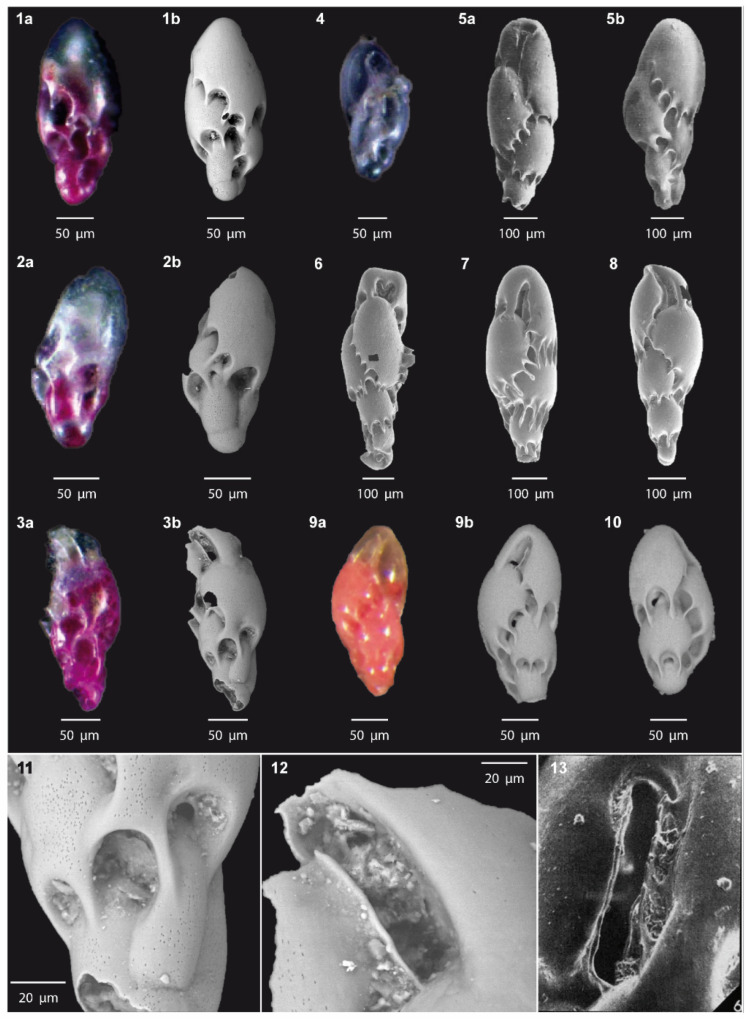
External test morphology of *V. fragilis* from NAS DPCR sampling stations and various locations documented in the literature. (**1**–**3**): Specimens of *V. fragilis* collected in 2019 from the S0 station (this study), photographed using a stereomicroscope equipped with a camera (a) and through ESEM (b). (**4**): *V. fragilis* specimen sampled in 2023 from the E1 station (this study). (**5**): aperture side (a) and back side (b) SEM images of *V. fragilis* holotype from Wellington Harbor, New Zealand originally captured by Grindell and Collen [84] and modified by Hayward et al. [168]. (**6**–**8**): ESEM images of *V. fragilis* specimens from Namako-ike, Kagoshima, Japan: (**6**), Walvis Bay, off Namibia (**7**), and Wellington Harbor, New Zealand (**8**), as reported by Tsuchiya et al. [94]. (**9**–**10**): *V. fragilis* specimens from port de Calais, France, photographed using a stereomicroscope with a camera (**9a**) and trough ESEM (**9b**,**10**) by Jorissen et al. [167]. (**11**): Close-up ESEM image of the finger-like sutures and characteristic arched sutural openings of a *V. fragilis* specimen collected in 2019 at the S0 station (this study). (**12**): Close-up ESEM image showing the slit-like aperture and loop-shaped tooth plate from a *V. fragilis* specimen sampled in 2019 at the S0 station (this study). (**13**): Close-up ESEM image of the slit-like aperture and loop-shaped tooth plate from the *V. fragilis* holotype from Wellington Harbor, New Zealand, originally documented by Grindell and Collen [84].

Living (stained) and dead (unstained) *V. fragilis* specimens were recovered in sediments collected at multiple NAS coastal stations located within the Italian LTER DPCR Site. Precisely, this taxon was initially found in the areas closest to the Po Delta, firstly at station S0 (~13 m depth) in 2019 samples (four living specimens; 2% of total living BF assemblages); then, at station S1-GB (~22 m depth) in 2021 samples (one dead specimen) until expanding southwards at station E1 (~10 m depth), Rimini offshore, in 2023 samples (two dead specimens) (Figure 1, Figure 2, Appendix A, Table A1). These *V. fragilis* occurrences here reported represent the first chronological record in the modern Adriatic Sea, documenting the introduction of this taxon into the central Mediterranean region. Details on species occurrences, abundances, and sampling dates are summarized in Table A1 (Appendix A), while bottom water environmental parameters are summarized in Table A2 (Appendix A).

### 5.2. Morphology of Virgulinella fragilis from NAdFC Samples

The external morphologies of *V. fragilis* specimens collected from the NAS DPCR stations (Figure 2, images 1–4) are closely similar to those from Port de Calais (France) documented by Jorissen et al. [167] (Figure 2, images 9–10). Both these morphotypes exhibit relatively smaller tests (~200 μm), a compressed test along the growth axis, and more flattened chambers and wider sutural arches when compared to the holotype from Wellington Harbor (New Zealand) described by Grindell and Collen [84] (Figure 2, images 5a,5b). In contrast, the first described and more recent *V. fragilis* specimens from Wellington Harbor (New Zealand), as well as specimens from Namako-ike (Kagoshima, Japan) and Walvis Bay (Namibia offshore) (Figure 2, images 6–8) [94], display larger (~600 μm), more elongated and tapered tests and chambers with slightly narrower arches, although Namibian specimens have more pronounced and elongated sutural bridges.

**Figure 3:**
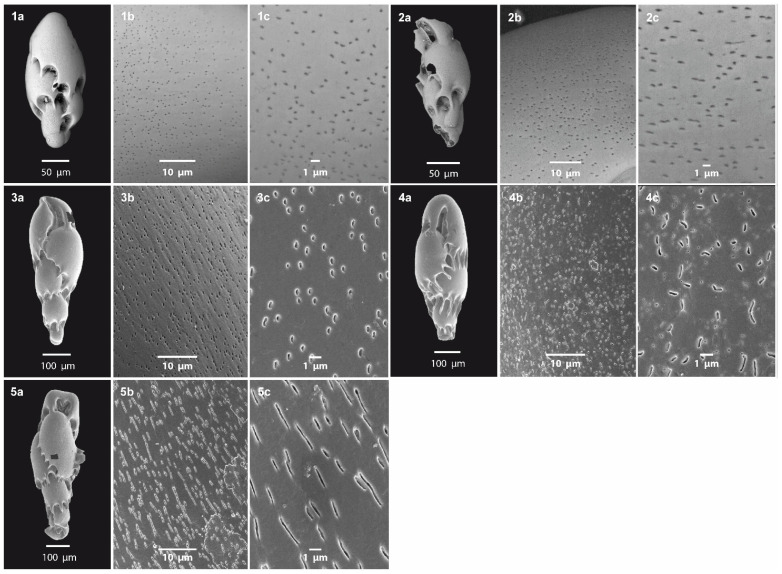
ESEM images of *V. fragilis* external morphology (**a**), shell surface with pore distribution and density on penultimate chamber (**b**), and close-up on pore shape (**c**). (**1**,**2**): *V. fragilis* specimens from NAS DPCR S0 sampling station (this study); (**3**): *V. fragilis* from Walvis Bay, off Namibia, from [94]; (**4**): *V. fragilis* from Wellington Harbor, New Zealand, from Tsuchiya et al. [94]; (**5**): *V. fragilis* from Namako-ike, Kagoshima, Japan, from Tsuchiya et al. [94].

Although we did not quantify PD as the number of pores within a defined area, visual comparison of scanning electron micrographs taken at the same magnification revealed that all the *V. fragilis* specimens in Figure 3 (images 1–5b) exhibited very similar pore densities. Differently, specimens of *V. fragilis* from the NAS DPCR site exhibit a variety of pore morphologies, displaying characteristics intermediate between those described by Tsuchiya et al. [94], for samples from Wellington Harbor and Walvis Bay (Figure 3, images 3b,3c,4b,4c). The PSh present two end-member forms, ranging from oval–nearly circular (Figure 3, images 1b,1c) to more elongated or irregularly shaped (Figure 3, images 2b,2c). Some of these elongated pores appear to result from the fusion of two or more individual pores. The oval–nearly circular pores have an average length of 0.294 μm (min: 0.283 μm; max: 0.327 μm), an average width of 0.276 μm (min: 0.217 μm; max: 0.318 μm), and a length/width ratio of 1.06. The elongated pores have an average length of 0.952 μm (min: 0.888 μm; max: 1.046 μm), an average width of 0.186 μm (min: 0.128 μm; max: 0.229 μm), and a length/width ratio of 5.12. Overall average values of PS derived from all the *V. fragilis* specimens analyzed in this study are the following: an average length of 0.615 μm, an average width of 0.225 μm, and a length/width ratio of 2.73.

### 5.3. Model Accuracy and Suitability Map

The model exhibited perfect accuracy and an optimal ability to discriminate between true and false instances, achieving an AUC value of 1.

**Figure 4:**
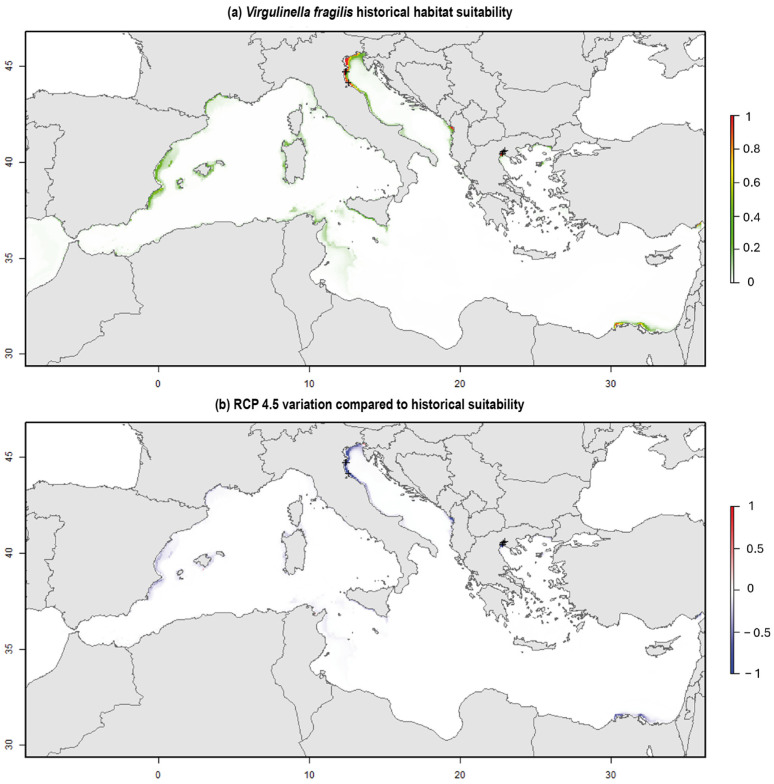
Map of HSMs *V. fragilis* in the Mediterranean basin based on (**a**) a historical period (2013–2023), and (**b**) the RCP 4.5 future climate change scenario (2050–2099).

The suitability map (Figure 4) identified areas with potential favorable conditions for the presence of *V. fragilis*. High suitability values (0.8–1), indicating very favorable habitat conditions, were observed along the North Adriatic coast. Suitability values significantly decreased both northward and southward within the basin, reaching values of approximately 0.4. A similar pattern was observed in the Thermaikos Gulf (Greece), the Albanian coast overlooking Tirana, and the Egyptian coast in front of the area of Alexandria.

The remaining Mediterranean coastline exhibited moderate suitability conditions, with values ranging between 0.4 and 0.6. Notable areas with medium suitability included the Murcia and Valenciana regions (Spain), the Gulf of Lion (France), the northeastern and western coast of Sardinia (Italy), and the southern coast of Sicily (Italy).

Projections based on the RCP 4.5 climate change scenario at the end of the century suggest a potential reversal of these favorable conditions, with a significant decline in suitability across all previously identified optimal habitats. The only area projected to experience an increase in suitability under future conditions is the Gulf of Trieste (northeastern Italy).

## 6. Discussion

### 6.1. Temporal and Spatial Patterns of Virgulinella fragilis Arrival in the Mediterranean and Adriatic Seas

Modern living and dead specimens of *V. fragilis* were first identified in Wellington Harbor, New Zealand, from silty to muddy sediments collected at depths of 16 to 31 m with normal bottom salinities (33.5 to 34.5 ‰) and temperatures seasonally ranging from 11 to 18° C [84]. Since then, living specimens of *V. fragilis* have been documented in recent sediments from various scattered regions worldwide, spanning from coastal to bathyal environments and diverse oceanic regimes. These regions include Australia [169], Peru [170,171], the coastal upwelling area off Namibia [94,172,173,174], the stratified waters of the Cariaco Basin in Venezuela [89,175,176], India [177,178], the oxygen minimum zone off Pakistan’s margin [91], and meromictic lakes and the marine domain in Japan [90,94]. Although, the occurrence of *V. fragilis* results in prevalence in the Caribbean and Gulf of Mexico areas [179,180].

More recently, this taxon has been detected as a genomic material in Belgian and Dutch waters [181,182], and as a specimen on the Atlantic French coast and its harbors [167,183], thus documenting the entrance of this taxon into the European seas and coastal areas. Relatively recent occurrences of *V. fragilis* were also recorded in the Mediterranean region and follow a clear temporal and spatial pattern. This potential foram-NIS was initially recorded within deep-sea sediments of the Marmara Sea at a depth of 329 m in 2014 [184]. Subsequently, in 2018, it was found in sublittoral sediments from the Thermaikos Gulf in the North Aegean Sea [166]. Currently, living specimens have been detected in sediments from the NAS DPCR Site, south of the Po Delta River at the shallow S0 station (this study, [145]), while the most recent occurrence, although within dead assemblages, was recorded in September 2023 further south in the northwestern Adriatic Sea, at the shallow E1 station, offshore Rimini [this study]. As the sole living specimens have been collected in S0, this may imply that the most favorable conditions for the *V. fragilis* presence (and proliferation?) in the western NAS are likely present at this station, but, unfortunately, we do not have further sampled material in subsequent years from S0 (Appendix A, Table A1).

The presence of a well-established *V. fragilis* population from both the Aegean Sea’s Gulf of Thermaikos [166] and the Maramara Sea—a semi-enclosed basin connecting the Aegean Sea and Levantine Basin to the Black Sea—where it is the second most abundant taxon in both the >63 µm and >125 µm fractions [184], suggest that within the eastern Mediterranean ecoregion, this potential foram-NIS may have spread prior to its recent documentation. Differently, considering the low recorded abundance of this taxon in the NAS DPCR Site, we hypothesize that this may represent an initial phase of a possible secondary spread likely related to oxygen-depleted and nutrient-rich conditions peculiar to the western NAS, and that, therefore, requires more in-depth investigations (see Section 6.4). Regardless of the marine environmental depth, *Virgulinella fragilis* is indeed typically found in hypoxic to near-anoxic environments rich in hydrogen sulfide, making it an effective proxy for oxygen-depleted or sulfide-rich habitats [90,91,92,93,94]. However, the absence of a consistent *V. fragilis* population throughout the years may be also related to different aspects such as seasonality or sampling depth.

Additionally, considering the temporal proximity between the occurrences recorded in the Thermaikos Gulf (2018) and the DPCR Site (2019) and the similar environmental condition of these two geographically separated areas, we hypothesize that dispersal may have occurred with the same introduction vector, implicating the same origin.

### 6.2. Putative Introduction Dynamic of Virgulinella fragilis in Mediterranean and Adriatic Seas

To properly classify a taxon as an introduced species/NIS, it is essential not only to consider its current distribution and its range of potential natural spreading but also to verify whether its introductions into a specific basin occurred in the geological past. This can only be achieved by scrutinizing the fossil record (e.g., [48,185]). Although fossils attributed to the genus *Virgulinella* have been identified in Oligocene, Miocene and Pliocene deposits across Europe (Belgium, Germany, Netherlands, Italy, Ciscaucasus) and North Africa (Algeria, Egypt) [186,187,188,189,190], these records do not correspond to the species *V. fragilis*. This absence in the geological history of the Mediterranean and Adriatic Seas leaves open the possibility of designating it as a NIS for these basins. We also verified whether fossils of *V. fragilis* have been documented in the stratigraphic record of the multiple sediment cores taken in the study area (e.g., [191] and reference therein), but we did not find any evidence.

Notably, numerous larger NIS but only a few foram-NIS have been documented in the Mediterranean and Adriatic seas. A total of 44 confirmed Mediterranean BF NIS, including tropical symbiont-bearing larger BF (LBF), smaller BF taxa, and two cryptogenic species, have been reported in a revised taxonomic assessment that lowers earlier counts from prior surveys ([48] and reference therein). As they generally enter the Mediterranean via the Suez Canal and then follow the counter-clockwise current pattern, most of these foram-NIS primarily established along the Levantine coasts in the Eastern Mediterranean Sea (41 taxa, ~93%), while only eight species have reached the Adriatic Sea. The distribution of tropical LBF in the Mediterranean and its ecoregions has been linked to the minimum winter sea surface temperatures, which act as a natural barrier controlling the LBF latitudinal distribution and currently limit their spread in temperate eastern Mediterranean waters and colder western regions influenced by Atlantic currents [79,192]. Under RCP4.5 projections, the warming climate is expected to enhance habitat suitability, leading to range expansions for LBF into the western and northern Mediterranean, including the northern Adriatic (e.g., [71,72,79]). In contrast, there is a significant lack of information regarding the rates of range expansions for smaller foram-NIS in the Mediterranean and Adriatic seas. Additionally, though recent genetic studies show that traditional taxonomy underestimates foraminifera diversity [193,194], Mediterranean and Adriatic foram-NIS remain still molecularly understudied.

Vectors of *V. fragilis* introduction in the Mediterranean and Adriatic seas may be multiple, and dispersal mechanisms may have occurred via both natural or anthropogenic means. Indeed, BF—and thus *V. fragilis*—may be rapidly dispersed in the marine environment through passive transport of their resting stages (propagules) and/or gametes. According to [195], the small food reserves of gametes limit their survival time, hindering long-distance dispersal, and Tsuchiya et al. [94] concluded that this dispersal mechanism appeared unlikely for the worldwide scattered *V. fragilis* populations, as well as a meroplanktonic life strategy (i.e., a temporary planktonic phase) [196]. Nonetheless, propagules have a great potential to disperse over long distances across seas and oceans, with microspheric propagules having greater chances than megalospheric propagules (due to the size of the proloculus) or small juveniles [92,196]. Additionally, by adopting the dormancy life strategy, cryptobiotic propagules may allow foraminifers to survive up to two years in degraded or unfavorable environments until conditions improve, becoming suitable for either growth or reproduction [92,197]. Moodley et al. [198] suggested that dormancy may have also contributed to the survival of post-propagule life stages for taxa such as *Nonionella* and *Stainforthia*—genetically related to *V. fragilis*—under experimentally induced anoxic conditions.

According to Stulpinaite et al. ([48] and references therein), the main Adriatic BF NIS introduction pathways and vectors include the following: (1) corridor, i.e., transport mainly, but not only, via the Suez Canal; (2) transport contaminant on animals, i.e., species transported by a host/vector, so far invoked only for *A. lobifera* [199]; (3) stowaway transport via ship/boat ballast water or hull fouling (e.g., [131]); and (4) stowaway transport attached to floating substrates (e.g., wood, seagrass leaves, rhizomes, algae, plastic waste) (e.g., [200]). Here, we will cover all the common and feasible options that may have led to the introduction of *V. fragilis* into the Mediterranean and Adriatic seas.

#### 6.2.1. Anthropic Means of Introduction

Due to the high abundance of *V. fragilis* recorded within the Aegean Sea’s Gulf of Thermaikos and the Marmara Sea [166,184], both close to the Levantine Basin, it is plausible to speculate that the first introduction of *V. fragilis* within the Mediterranean may have occurred via the route of the Suez Canal. Unlike trends and patterns observed in Europe and globally, where canals typically rank lower in importance with respect to shipping (via ballast waters or hull fouling), the Suez Canal serves as the primary pathway (~54%) for the unintentionally introduced marine NIS in the Mediterranean, particularly affecting the eastern Mediterranean Levantine Basin [28,31,32,201,202]. Nonetheless, there is no evidence at present of the presence of *V. fragilis* from the southern Levantine Basin. Additionally, as *V. fragilis* has not been described as a thermophilic taxon, and due to the extensive monitoring of NIS/IAS in the Levantine Basin, along with the fact that the highest number of foram-NIS has been reported there (e.g., [48] and references therein), it is improbable, though not impossible, that the presence of *V. fragilis* in this region has gone unnoticed. Consequently, we suggest that the introduction of *V. fragilis* via the Suez Canal route is unlikely.

Alternatively, although not yet documented, it cannot be excluded that a relict population of *V. fragilis* was already present in the geological past of the Black Sea and has continued to inhabit this area unnoticed up to nowadays. If this is the case, it is possible that, in a relatively recent period, rapid dispersal of this species from the Black Sea to the Marmara and subsequently to the Adriatic basins may have occurred through marine shipping routes (stowaway transport via hull fouling or ballast water). As a matter of fact, the Black Sea possesses environmental seabed conditions that match the ecological preferences of *V. fragilis*. Indeed, the Black Sea stands as the largest semi-enclosed meromictic basin globally (i.e., deep waters do not mix with the upper well-oxygenated waters), featuring a persistent anoxic layer that lies beneath depths of 100–150 m [203,204]. This anoxic zone is marked by elevated concentrations of hydrogen sulfide (H_2_S), which restricts the majority of the benthic life to the continental Black Sea shelf areas, but can provide a perfect environment for the proliferation of *V. fragilis*, which is tolerant to nearly anaerobic conditions and is adapted to sulfide-enriched habitat [89,90,91].

Multiple studies highlight that the majority of the NIS unintentional introductions can be attributed to shipping trade [131,205,206,207,208]. In particular, the Mediterranean Sea is among the world’s busiest waterways, accounting for 15% of global shipping activity [209,210]. The major transport axis, mainly for oil transport, is from east to west, connecting the eastern passages of the Straits of the Dardanelles with the western Straits of Gibraltar. This axis passes between Sicily and Malta and closely follows the coasts of Tunisia, Algeria, and Morocco, with traffic branches extending to unloading terminals in Greece and the northern Adriatic harbors [209,211].

Considering the following factors: (i) maritime transport is the primary mechanism for introducing species into both the North Aegean and Adriatic seas [31,32,131]; (ii) marine shipping provides a connection between all three locations where *V. fragilis* specimens were recently found in the Mediterranean area; and (iii) both the Thermaikos Gulf and the DPCR site are situated near major harbor areas while the Marmara Sea is a passage for a main shipping route [209,211], we propose that stowaway transport could be the preferred pathway for the dispersal of this taxon in the Mediterranean ecoregions, including the study area. Notably, both Grindell and Collen [84], and Delliou et al. [166], suggested that ballast water, which involves the same shipping mechanism, is the most likely vector for the introduction of *V. fragilis* into Wellington Harbor and Thermaikos, respectively. Although hull fouling has been identified as the most common vector for marine alien species introduced into European seas so far [28], we lack sufficient evidence to determine whether *V. fragilis* was introduced into the Mediterranean and Adriatic seas via hull fouling or ballast water. However, according to our hypothesis on the shipping vector, *V. fragilis* may be thus considered an effective NIS [17,19,26]. Additionally, due to the high morphological resemblance of France and NAS *V. fragilis* specimens, and considering that Pavard et al. [183] suggest that stowaway transport is the most likely introduction pathway responsible for the arrival of various foram-NIS, including *V. fragilis*, in French harbors and adjacent coastal areas, this validates our hypothesis on the *V. fragilis* arrival mode in the NAS.

Although the shipping mechanism may explain the spread within the Mediterranean Sea, this process alone does not reveal the *V. fragilis* propagules’ origin. The widespread, though patchy, distribution of *V. fragilis* makes difficult to definitively determine whether the Mediterranean population arrived via marine shipping from the Black Sea, the Atlantic, or the Indo-Pacific oceans. Furthermore, there is no consensus in the literature regarding the source area for this taxon. Hayward et al. [168] suggested that *V. fragilis* likely originated from the Caribbean due to its widespread distribution in that area. Differently, Delliou et al. [166] and Pavard et al. [183] proposed an Indo-Pacific origin. Considering the low genetic variability of *V. fragilis* populations from the different regions of the world [94], neither genomic analysis may help in this.

Aquaculture and shellfish farming may also represent a significant pathway for introducing and spreading NIS into the NAS DPCR site and stations located in the proximity of multiple coastal lagoons exploited for these activities [212]. The Venice Lagoon, a hotspot for marine and brackish NIS in the Adriatic Sea, frequently sees aquaculture—rather than shipping—as the primary vector for NIS introductions. This lagoon serves as both a sink and source for NIS dispersal across Mediterranean sites, including the Po Delta lagoons such as Goro [130], located nearby the DPCR S0 station, where living *V. fragilis* specimens have been collected. Di Blasio et al. [212] reviewed literature data to assess the impact of shellfish culture on NIS introduction across multiple groups of taxa, identifying oyster farming as the dominant source of alien species introductions, followed by mussels and clams. However, their compiled record of introduced taxa did not report any foraminifera. Additionally, as in the Po Delta lagoons shell farms are mainly based on seeds from a local nursery and natural spat [212,213], we exclude this vector as a main driver for the arrival of *V. fragilis* in the NAS DPCR site.

#### 6.2.2. Natural Means of Introduction

The passive transport of propagules through marine currents is one of the most feasible as the resuspension of fine-grained sediments occurs easily in shallow waters. Populations of *V. fragilis* living at shallow depths, such as those from the Thermaikos Gulf and from the DPCR area, may have therefore been resuspended in the water column and been passively transported to distant locations by currents. Considering the Mediterranean circulation pattern connecting the Aegean Sea with the NAS and the cyclonic surface circulation of the latter connecting the eastern to western coastal areas, this mechanism may be relevant to explain the secondary introduction of *V. fragilis* in the DPCR Adriatic site from the Thermaikos Gulf [214]. Nonetheless, recently published studies documenting BF assemblage composition from sampling conducted during the last decade (from 2010 to today) on the eastern Adriatic banks influenced by the EACC did not document the presence of *V. fragilis* in these areas (e.g., [215,216,217,218]). Although it cannot be excluded that this lack of presence may be related to monitoring protocols [51], which recommend analyzing only live BF tests from the >125 μm fraction, potentially neglecting small and elongated *V. fragilis* specimens, this spread route cannot be sufficient to justify the former presence of *V. fragils* specimens from the deep Marmara Sea. Therefore, we exclude this as the main dispersal mechanism to explain the arrival of *V. fragilis* in both the NAS and Mediterranean Sea. In contrast, as the DPCR coastal area is significantly influenced by the WACC circulation, propagule transport via this marine current is the most plausible vector that may have been responsible for the southward dispersal of *V. fragilis* from S0 to the E1 stations.

Another natural means of passive transportation that may have been responsible for the *V. fragilis* introduction into the NAS DPCR site as well as in the Thermaikos Gulf is passive transport through shorebirds, which may act as natural vectors for foraminifera dispersal between shallow-water habitats. Shorebirds can indeed potentially deposit muddy sediments containing living foraminifera or their propagules via their feet or feathers after landing on the water’s surface or during fishing activity. If the environmental conditions are appropriate upon reaching the sea floor, these organisms can colonize the sediment. Multiple studies propose avian transport as a dispersal vector for foraminifera. Through this means, Resig [219] justified the introduction of foraminifera into the isolated Hawaiian Salt Lake, while Tsuchiya et al. [94] explained the introduction of *V. fragilis* into the meromitic Namako-ike Lake in Japan.

Both the NAS DPCR site as well as the Thermaikos Gulf [220,221] are close to coastal wetland and lagoon protected areas that are important for African and American migratory birds, as a stopover or reproductive site (e.g., [222,223]). Interestingly, the Namibia Walvis Bay wetlands and lagoons, where an abundant *V. fragilis* population was documented [174], are known as a major African site supporting migratory seabirds [224]. Nonetheless, avian transport cannot explain the former presence of *V. fragilis* in the deep Marmara Sea, so we also exclude this vector from those responsible for the dispersal of *V. fragilis* in the Mediterranean area.

### 6.3. Virgulinella fragilis External Morphology and Porosity as a Tool to Infer Sea-Bottom Oxygenation Levels and Changes

BF adapted to oxygen-depleted habitats often exhibit small, flattened, elongated, or thin-walled tests [225,226,227,228]. Since pores facilitate substance exchange with the surrounding environment, increased porosity enhances oxygen intake efficiency—a crucial metabolic requirement under low-oxygen conditions—and the absorption of organic substances for feeding and respiration [229,230]. As a result, BF may exhibit test plasticity, adjusting their porosity, particularly varying PD and PS, between well-oxygenated and oxygen-depleted environments [229,231,232]. PD inversely correlates with bottom dissolved oxygen (DO), being 36% higher in hypoxic conditions (DO < 3 mg/L) [231,233], while PS shows a more variable relationship with DO [234].

*Virgulinella fragilis* is characterized by typical morphologies of low-oxygen-tolerant BF such as an elongated shape and a very thin-walled fragile tests, with the latter likely the reason for the attributed name. Although external morphologies and the genetic pool are the same, the porosity of *V. fragilis* differs between localities (Figure 3), thus showing plasticity, likely related to environmental conditions, such as differences in oxygen or sulfide concentrations [93,94]. Interestingly, the porosity of *V. fragilis* is not only related to PD and PS but also to the pore shapes (PSh), which become elongated and/or interconnected in highly hypoxic environments ([O2] < 62.5 μM, i.e., Namako-ike Lake, Japan) while they are smaller and oval in better-oxygenated sediments ([O2] > 62.5 μM, i.e., Wellington, New Zealand) [94].

Porosity in specimens of *V. fragilis* from the study area (Figure 3, images 1–2) exhibits intermediate characteristic in PSh, and PS when compared to those described by Tsuchiya et al. [94], from Wellington Harbor and Namibia (Figure 3, images 3–4). Oval-shaped pores (Figure 3, images 1a–c) correlate well with environmental parameters recorded at the DPCR stations, where DO concentrations indicate a relatively oxygenated seafloor, not reaching hypoxic conditions (Appendix A, Table A2). The same PSh has indeed been observed in recent sediments from Wellington Harbor (Figure 3, images 3a–c), where the hypoxia noted in the 1960s and 1970s [235,236] is currently absent and oxic conditions prevail in the sediment’s surface layer, although this shift has led to a reduction in the *V. fragilis* population [94]. In contrast, the elongated and irregular pores (Figure 3, images 2a–c) are more similar to those of the highly hypoxic Walvis Bay upwelling area (Figure 3, images 4a–c, Appendix A, Table A2) and may suggest a temporal decrease in DO within the DPCR area. As benthic foraminifera have a relatively short life span, typically ranging from weeks to several months [237], it is thus possible that the penultimate chambers of different *V. fragilis* specimens have developed in environments with different DO concentrations. Therefore, we interpret this intermediate DPCR *V. fragilis* porosity as indicative of periodic oxygen deficiency.

**Figure 5:**
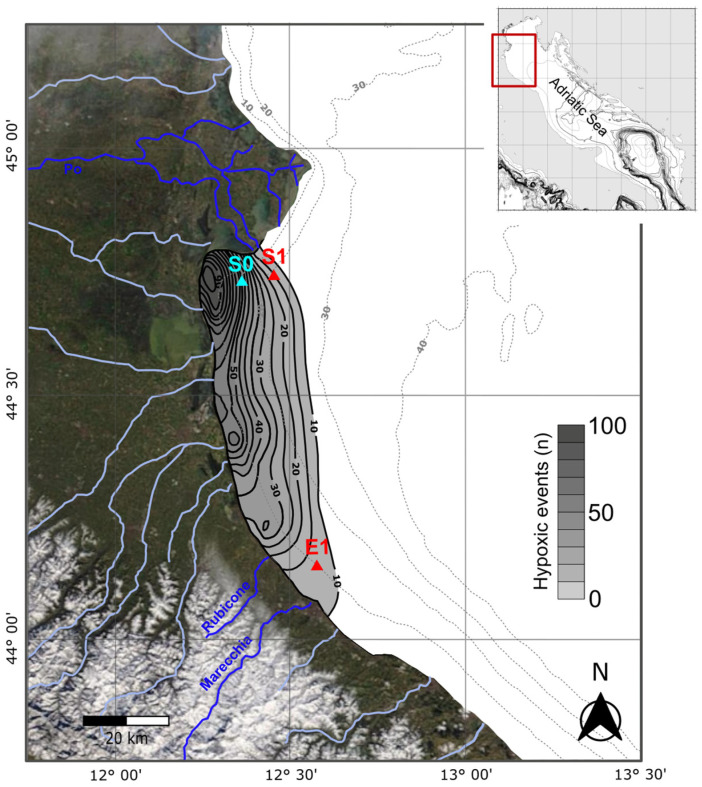
Satellite image with location of the S0, S1-GB, and E1 LTER DPCR sampling stations (triangles) showed in the frame of the NAS hypoxic pattern from [238]. Other information is described in the Figure 1 caption.

In the Po prodelta, seasonal oxygen deficiency has been extensively documented [120,121,238], thereby supporting our hypothesis. According to the literature, areas most affected by hypoxic events are those located south of the Po Delta and the inshore locations, due to the coupled effects of river loads and morpho-hydrodynamics (Figure 5). Notably, the sole living specimens from the DPCR site have been sampled at the S0 station, where hypoxic events are more frequent, with it being located close to the inshore area south of the Po Delta (Figure 5) [238]. Previous studies linked the NAS-related seasonal hypoxia to eutrophy and organic matter degradation that mainly occurrs during periods of sediment starvation and water stratification [120,121,239]. However, Alvisi and Cozzi [238] observed a decline in the trophic level of the NAS over recent decades. This shift has led to a more irregular trend in hypoxia, which is now influenced by stable weather patterns and wind conditions. These conditions can reduce circulation, trapping river plumes near the coast due to winds like the Bora and Scirocco, or trigger the upwelling of offshore hypoxic waters along the coastline. Irregular runoff and warming temperatures likely caused hypoxia to spread year-round and increased the frequency of severe events previously confined to summer [238].

As hypoxia in the NAS may impact the entire water column and the first centimeters of the sea bottom sediments may be partially or completely oxygenated depending, among other factors, on the sediment/water interface DO concentrations, our sampling depth (0–1 cm) supports our hypothesis on *V. fragilis* intermediate porosity as indicative of periodic oxygen deficiency at the DPCR site. Nonetheless, most BF infaunal taxa, such as *V. fragilis*, exhibit mobility during their life cycle within sediment layers [240,241,242]. Variations across sediments’ vertical profiles may reflect ecological preferences specific to each species, as well as their adaptive responses to environmental factors like food availability and DO levels [243,244,245,246]. While one study documented occurrences at 9–11 cm depth [174], the majority of the few published papers documenting the *V. fragilis* vertical distribution in sediments according to depth indicate that occurrences of this taxon are recorded within the upper sediment layers (0–1.5 cm) [171,173,184]. This further validates our proposed linkage of the intermediate porosity patterns observed in *V. fragilis* to episodic oxygen-depleted conditions at the NAS DPCR site.

### 6.4. Historical and Future Ranges of Habitat Suitability for V. fragilis in the Mediterranean

The application of Habitat Suitability Models (HSMs) enables the identification of potential areas where the species of interest may be found due to the presence of suitable environmental conditions (Figure 4a). Moreover, by projecting the model under climate change scenarios, it is possible to gain insights into the potential spread of NIS under changed environmental conditions (Figure 4b). This, in turn, can support the implementation of targeted monitoring protocols to assess the species presence and absence.

Regarding the MaxEnt model applied in this study, the calculated AUC metric indicated a perfect ability to distinguish between true positive and false positive rates. However, we underline that this result may be influenced by the limited number of occurrences used in the analysis, which may not sufficiently capture the ecological complexity governing the presence and absence of *V. fragilis* in the Mediterranean Basin.

Our model suggests that the primary factor influencing the presence of *V. fragilis* in the Mediterranean Basin is the bacterial concentration expressed as NO_3_. Besides the DO, the ecology of *V. fragilis* is strongly related also to the presence of specific bacterial endosymbionts (see Section 2), including the Deltaproteobacterium (Desulfobacteraceae), able to grow chemoautotrophically [247], which may enable this taxon to activate denitrification processes and consequently adsorb the necessary amount of nitrogen [93,94,248], a finding that reinforces the model’s validity. Furthermore, this may support the hypothesis outlined by Woehle et al. [248] regarding the potential denitrifying capabilities of *V. fragilis*. Notably, *V. fragilis* has often been found in association with bacterial or microbial mats in both coastal and deep-water sediments [91,174,184].

Moreover, most of the areas denoting suitable habitat conditions for *V. fragilis* (Figure 4a) (e.g., DPCR site, Thermaikos Gulf, Alexandria, Tirana coastal areas, Murcia, Valenciana, Gulf of Lion) are located near major Mediterranean river mouths (Nile, Po, Rhône, Ebro, Júcar, Segura), suggesting a link between *V. fragilis* presence and freshwater and nutrient inputs, including nitrate, from these rivers (e.g., [109,110,112,249]). Unfortunately, except for the Thermaikos Gulf and the DPCR site, recent studies (post-2018) have not reported *V. fragilis* in these areas (e.g., [250,251]). This absence may be due to monitoring protocols [51], which recommend analyses only on BF living assemblages in the >125 μm fraction, potentially overlooking small, elongated *V. fragilis* specimens. Remarkably, we observed BF assemblages from the >63 μm fraction.

Although an analysis conducted at the Mediterranean Basin scale may yield coarse results, it is important to acknowledge that, as this represents the first study on this species in a region where *V. fragilis* had not previously spread or yet established. For that reason, we believe it is essential to carry out this research, to further characterize the presence of this foram-NIS in our area of investigation in order also to provide guidance on where future sampling efforts should be directed. Furthermore, the registration of new occurrences in shared distributed databases will facilitate more targeted and narrow investigations in the Mediterranean Basin, enhancing the identification of correlations between past and present species occurrence with environmental trends.

According to the consideration above, we strongly encourage the expansion of monitoring efforts in order to verify the presence of *V. fragilis*, particularly in the areas identified as suitable by the model. This would allow for validation of the model’s predictions and provide additional reliable data, which will be needed to further refine and improve future analyses.

Considering the known ecological resilience of *V. fragilis* to oxygen-depleted conditions [90,94,252] and that our computed model was calibrated using dissolved oxygen values measured at the water–sediment interface, which may not fully capture hypoxic conditions that could be present within the sediment itself, we suggest verifying the presence of this taxon in Mediterranean regions where these conditions prevail.

Because of the non-indigenous characteristic of *V. fragilis*, it is appropriate to conduct investigations under climate change scenarios to understand species behavior and potential spread in changed environmental conditions. Indeed, thanks to datasets forecasting the evolution of the biogeochemical parameters in future conditions, we are capable of estimating where species might occur and if invasive behavior occupying local ecological niches is expected. Habitat Suitability Models under climate change conditions confirm themselves as being very suitable tools in searching for potential changes in biodiversity rates and for investigating species evolution and distribution according to changed environmental conditions. Regarding the RCP 4.5 future climate change scenario (Figure 4b), the decrease in suitable conditions in areas where *V. fragilis* is currently present suggests that this species is unlikely to become invasive in the Mediterranean Basin.

## 7. Summary and Conclusions

Recent cataloguing and taxonomic revision efforts, focused on the CNR-ISMAR Foraminifera Collection, as part of DiSSCo-ITINERIS activities, led to the identification of *Virgulinella fragilis*, a potential BF NIS, in samples from the NAS DPCR coastal sediments.

After evaluating both natural and anthropogenic species introduction pathways, and based on *V. fragilis* temporal and spatial patterns of arrival in the Marmara, North Eagan, and Adriatic seas, we propose that maritime shipping stowaway transport is the most likely dispersal mechanism for *V. fragilis* in the Mediterranean. Thus, we conclude that *V. fragilis* is an effective NIS due to its spread via shipping, an anthropic means. However, due to a lack of direct evidence, we cannot distinguish whether hull fouling or ballast water is the primary vector. Morphological analysis of *V. fragilis* from the NAS DPCR site revealed similarities to specimens from Port de Calais (France), with smaller sizes and flattened chambers compared to the Wellington Harbor holotype. DPCR specimens exhibited both oval-shaped pores, indicating oxic conditions, which were supported by environmental data showing a relatively oxygenated seabed and elongated, irregular pores, suggesting periodic oxygen deficiency, consistent with seasonal hypoxia in the Po prodelta.

In order to assess the possible trend of future spread of *V. fragilis*, an attempt was made to analyze the recent and future distribution, correlating its presence with environmental factors. Our Habitat Suitability Model based on historical data suggests that the bacterial concentration expressed as nitrate is a primary driver of *V. fragilis* presence in the Mediterranean, likely linked to riverine nutrient input. However, our RCP 4.5 climate change scenario predicts a decline in habitat suitability, making an invasive spread of *V. fragilis* in the Mediterranean unlikely.

This result highlights the importance of long-term biomonitoring and review of scientific archives to ensure early detection of NIS and IAS as well as the possibility to better understand origins and invasion dynamics. These actions are particularly relevant in areas which are known to be strongly impacted by anthropic activities and climatic and environmental changes, such as the North Adriatic and the Mediterranean.

Analyses on BF assemblages will be performed in the future at station S0 and on related depth transects in the Po prodelta area to verify the presence of this taxon and its depth and vertical distribution. This will allow us to better comprehend the ecological preferences of *V. fragilis*, enabling the refinement of suitability models.

## Data Availability

The original contributions presented in this study are included in Appendix A. Further inquiries can be directed to the corresponding author(s).

## Appendix A

**Table A1:** Sampling events at the LTER DPCR stations and *V. fragilis* occurrence details (highlighted in bold).

Type of Activity	Station	Date of Sampling	Latitude (N)	Longitude (E)	Depth (m)	*Virgulinella fragilis* Specimens (n)	Type of Specimens
LTER-ANOC16 Cruise	E1	28/04/2016	44°08.53	12°34.37	10.5		
LTER-ANOC16 Cruise	S1-GB	29/04/2016	44°44.32	12°27.10	22.5		
LTER-ANOC16 Cruise	S2	29/04/2016	44°43.91	12°30.22	26		
LTER-ANOC16 Cruise	S3	29/04/2016	44°44.18	12°37.84	31		
Marine strategy-Adriatic Sea Cruise	E1	14/12/2016	44°08.65	12°34.37	10.5		
Marine strategy-Adriatic Sea Cruise	S1-GB	15/12/2016	44°44.16	12°27.36	22.5		
ordinary maintenance	S1-GB	21/07/2016	44°44.31	12°27.16	22.5		
INTERNOS17 Cruise	S1-GB	15/03/2017	44°44.31	12°27.26	22.5		
INTERNOS17 Cruise	E1	19/03/2017	44°08.35	12°34.19	10.5		
ordinary maintenance	S1-GB	13/06/2017	44°44.31	12°27.26	22.5		
ordinary maintenance	E1	14/12/2017	44°08.60	12°34.20	10.5		
ordinary maintenance	E1	11/04/2018	44°08.60	12°34.2	10.5		
ordinary maintenance	S1-GB	17/05/2018	44°44.31	12°27.26	22.5		
ordinary maintenance	E1	30/06/2018	44°08.60	12°34.20	10.5		
ordinary maintenance	S1-GB	19/07/2018	44°44.31	12°27.26	22.5		
ordinary maintenance	E1	09/09/2018	44°08.60	12°34.20	10.5		
ordinary maintenance	S1-GB	24/10/2018	44°44.31	12°27.26	22.5		
ordinary maintenance	E1	04/12/2018	44°08.60	12°34.20	10.5		
INTERNOS19 Cruise	S1-GB	21/02/2019	44°44.27	12°27.27	20.2		
INTERNOS19 Cruise	S0	21/02/2019	44°44.00	12°21.929	12.5		
INTERNOS19 Cruise	S2	21/02/2019	44°43.98	12°30.404	24.3		
INTERNOS19 Cruise	S3	21/02/2019	44°44.17	12°37.856	29.6		
INTERNOS19 Cruise	S3	26/02/2019	44°44.20	12°37.684	18.2		
INTERNOS19 Cruise	S2	26/02/2019	44°43.94	12°30.392	29.8		
INTERNOS19 Cruise	S1-GB	26/02/2019	44°44.40	12°26.864	24.7		
**INTERNOS19 Cruise**	**S0**	**26/02/2019**	**44°43.90**	**12°22.034**	**20.2**	**4 * (2%)**	**living**
INTERNOS19 Cruise	E1	27/02/2019	44°08.54	12°34.809	13.1		
ordinary maintenance	S1-GB	25/03/2019	44°44.31	12°27.26	22.5		
ordinary maintenance	S1-GB	12/07/2019	44°44.31	12°27.26	22.5		
ordinary maintenance	E1	02/06/2021	44°08.60	12°34.20	10.5		
ordinary maintenance	S1-GB	27/06/2021	44°44.31	12°27.26	22.5		
ordinary maintenance	E1	19/09/2021	44°08.60	12°34.20	10.5		
**ordinary maintenance**	**S1-GB**	**23/12/2021**	**44°44.31**	**12°27.26**	**22.5**	**1**	**dead**
ordinary maintenance	S1-GB	25/03/2022	44°44.31	12°27.26	22.5		
ordinary maintenance	E1	21/05/2022	44°08.60	12°34.20	10.5		
ordinary maintenance	S1-GB	13/09/2022	44°44.31	12°27.26	22.5		
ordinary maintenance	E1	03/07/2022	44°08.60	12°34.20	10.5		
ordinary maintenance	S1-GB	19/10/2022	44°44.31	12°27.26	22.5		
ordinary maintenance	S1-GB	19/07/2023	44°44.31	12°27.26	22.5		
ordinary maintenance	S1-GB	24/08/2023	44°44.31	12°27.26	22.5		
**ordinary maintenance**	**E1**	**12/09/2023**	**44°08.60**	**12°34.20**	**10.5**	**2**	**dead**
ordinary maintenance	S1-GB	17/10/2023	44°44.31	12°27.26	22.5		
ordinary maintenance	S1-GB	11/07/2024	44°44.31	12°27.26	22.5		
ordinary maintenance	E1	27/07/2024	44°08.60	12°34.20	10.5		
ordinary maintenance	S1-GB	01/10/2024	44°44.31	12°27.26	22.5		
ordinary maintenance	S1-GB	13/12/2024	44°44.31	12°27.26	22.5		

* The percentage in brackets refers to the total living BF assemblages.

**Table A2:** Environmental parameters at the LTER DPCR sampling stations.

Sampling Localities	Sampling Station	Latitude	Longitude	Depth (m)	Environment	T * (°C)	Salinity (psu)	DO * (μmol/L)
Po River Delta, NAS (Italy)	S0	44°43.98 N	12°21.90 E	13.5	Prodelta	8.76	35.64	620.0
Po River Delta, NAS (Italy)	S1-GB	44°44.46 N	12°27.36 E	22.5	Prodelta	12.76	37.77	435.0
Costa Romagnola, NAS (Italy)	E1	44°8.58 N	12°34.26 E	10.5	Coastal	25.27	36.58	555.0

* Values collected at the sediment surface.

## References

[1] Cardinale B.J., Duffy J.E., Gonzalez A., Hooper D.U., Perrings C., Venail P., Narwani A., Mace G.M., Tilman D., Wardle D.A. (2012). Biodiversity loss and its impact on humanity. Nature.

[2] Halpern B.S., Longo C., Lowndes J.S.S., Best B.D., Frazier M., Katona S.K., Kleisner K.M., Rosenberg A.A., Scarborough C., Selig E.R. (2015). Patterns and emerging trends in global ocean health. PLoS ONE.

[3] Halpern B.S., Frazier M., Potapenko J., Casey K.S., Koenig K., Longo C., Lowndes J.S., Rockwood R.C., Selig E.R., Selkoe K.A. (2015). Spatial and temporal changes in cumulative human impacts on the world’s ocean. Nat. Commun..

[4] Stock A., Crowder L.B., Halpern B.S., Micheli F. (2018). Uncertainty analysis and robust areas of high and low modeled human impact on the global oceans. Conserv. Biol..

[5] Díaz S., Settele J., Brondízio E.S., Ngo H.T., Agard J., Arneth A., Balvanera P., Brauman K.A., Stuart H., Butchart M. (2019). Pervasive human-driven decline of life on Earth points to the need for transformative change. Science.

[6] Cheung W.W.L., Lam V.W.Y., Sarmiento J.L., Kearney K., Watson R., Pauly D. (2009). Projecting global marine biodiversity impacts under climate change scenarios. Fish Fish..

[7] Butchart S.H.M., Walpole M., Collen B., van Strien A., Scharlemann J.P.W., Almond R.E.A., Baillie J.E.M., Bomhard B., Brown C., Bruno J. (2010). Global biodiversity: Indicators of recent declines. Science.

[8] Steffen W., Rockström J., Richardson K., Fetzer I., Bennett E.M., Biggs R., Carpenter S.R., De Vries W., De Wit C.A., Folke C. (2015). Planetary boundaries: Guiding human development on a changing planet. Science.

[9] Lotze H.K., Tittensor D.P., Bryndum-Buchholz A., Eddy T.D., Cheung W.W.L., Galbraith E.D., Barrier N., Bianchi D., Blanchard J.L., Bopp L. (2019). Global ensemble projections reveal trophic amplification of ocean biomass declines with climate change. Proc. Natl. Acad. Sci. USA.

[10] Jouffray J.B., Norström A.V., Nyström M., Folke C., Moberg F. (2020). Marine biodiversity loss and its cascading effects. Nat. Sustain..

[11] Gissi E., Manea E., Mazaris A.D., Fraschetti S., Almpanidou V., Bevilacqua S., Coll M., Guarnieri G., Lloret-Lloret E., Pascual M. (2021). A review of the combined effects of climate change and other local human stressors on the marine environment. Sci. Total Environ..

[12] Brondizio E.S., Settele J., Díaz S., Ngo H.T., IPBES (2019). Global Assessment Report on Biodiversity and Ecosystem Services of the Intergovernmental Science-Policy Platform on Biodiversity and Ecosystem Services.

[13] Lee H., Romero J., IPCC (2023). Climate Change 2023: Synthesis Report.

[14] Vitousek P.M., D’Antonio C.M., Loope L.L., Westbrooks R. (1996). Biological invasions as global environmental change. Am. Sci..

[15] Ojaveer H., Galil B.S., Campbell M.L., Carlton J.T., Canning-Clode J., Cook E.J., Davidson A.D., Hewitt C.L., Jelmert A., Marchini A. (2015). Classification of non-indigenous species based on their impacts: Considerations for application in marine management. PLoS Biol..

[16] Ojaveer H., Galil B.S., Carlton J.T., Alleway H., Goulletquer P., Lehtiniemi M., Marchini A., Miller W., Occhipinti-Ambrogi A., Peharda M. (2018). Historical baselines in marine bioinvasions: Implications for policy and management. PLoS ONE.

[17] Convention on Biological Diversity (CBD) (2011). Aichi Target 9: Invasive Alien Species. EU Strategic Plan for Biodiversity 2011–2020.

[18] European Parliament and Council (2014). Regulation (EU) No 1143/2014 on the prevention and management of the introduction and spread of invasive alien species. Off. J. Eur. Union.

[19] EU (2014). Report from the Commission to the European Parliament and the Council. The mid-term review of the EU Biodiversity Strategy to 2020. European Commission Report.

[20] United Nations Transforming Our World: The 2030 Agenda for Sustainable Development [UN A/RES/70/1]. https://undocs.org/A/RES/70/1.

[21] European Commission (2020). EU Biodiversity Strategy for 2030: Bringing Nature Back into Our Lives.

[22] European Parliament and Council (2008). Directive 2008/56/EC of the European Parliament and of the Council of 17 June 2008 establishing a framework for community action in the field of marine environmental policy (Marine Strategy Framework Directive). Off. J. Eur. Union.

[23] European Parliament and Council (2017). Directive (EU) 2017/845 of the European Parliament and of the Council of 17 May 2017 amending Directive 2008/56/EC of the European Parliament and of the Council. Off. J. Eur. Union.

[24] European Parliament and Council (2017). Directive (EU) 2017/848 of the European Parliament and of the Council of 17 May 2017 on the assessment of good environmental status and the establishment of environmental targets under the Marine Strategy Framework Directive. Off. J. Eur. Union.

[25] Technical Group 2 (TG2) (2020). Impact of Biological Pollution and Bi-Invasion on Ecosystems. Biodiversity and Invasive Species: Global Challenges and Management Approaches.

[26] EU Commission Decision (EU) 2017/848 of 17 May 2017: Glossary. https://mcc.jrc.ec.europa.eu/main/dev.py?N=20&O=119&titre_chap=D2+Non-indigenous+species#201671114752.

[27] Elton C.S. (1958). The Ecology of Invasions by Animals and Plants.

[28] Katsanevakis S., Zenetos A., Belchior C., Cardoso A.C. (2013). Invading European Seas: Assessing Pathways of Introduction of Marine Aliens. Ocean Coast. Manag..

[29] Ojaveer H., Olenin S., Narščius A., Florin A.-B., Ezhova E., Gollasch S., Jensen K.R., Lehtiniemi M., Minchin D., Normant-Saremba M. (2017). Dynamics of Biological Invasions and Pathways Over Time: A Case Study of a Temperate Coastal Sea. Biol. Invasions.

[30] Pergl J., Brundu G., Harrower C.A., Cardoso A.C., Genovesi P., Katsanevakis S., Lozano V., Perglová I., Rabitsch W., Richards G. (2020). Applying the Convention on Biological Diversity Pathway Classification to alien species in Europe. NeoBiota.

[31] Zenetos A., Gofas S., Verlaque M., Çinar M.E., García Raso J.E., Bianchi C.N., Morri C., Azzurro E., Bilecenoglu M., Froglia C. (2010). Alien species in the Mediterranean Sea by 2010. A contribution to the application of European Union’s Marine Strategy Framework Directive (MSFD). Part I. Spatial distribution. Medit. Mar. Sci..

[32] Zenetos A., Gofas S., Morri C., Rosso A., Violanti D., Garcia Raso J.E., Cinar M.E., Almogi-Labin A., Ates A.S., Azzurro E. (2012). Alien species in the Mediterranean Sea by 2012. A contribution to the application of European Union’s Marine Strategy Framework Directive (MSFD). Part 2. Introduction trends and pathways. Mediterr. Mar. Sci..

[33] Rilov G., Galil B., Rilov G., Crooks J.A. (2009). Marine bioinvasions in the Mediterranean Sea—History, distribution and ecology. Biological Invasions in Marine Ecosystems.

[34] Galil B.S. (2007). Loss or gain? Invasive aliens and biodiversity in the Mediterranean Sea. Mar. Pollut. Bull..

[35] Tsirintanis K., Azzurro E., Crocetta F., Dimiza M., Froglia C., Gerovasileiou V., Langeneck J., Mancinelli G., Rosso A., Stern N. (2022). Bioinvasion impacts on biodiversity, ecosystem services, and human health in the Mediterranean Sea. Aquat. Invasions.

[36] Edelist D., Rilov G., Golani D., Carlton J., Spanier E. (2013). Restructuring the Sea: Profound shifts in the world’s most invaded marine ecosystem. Divers. Distrib..

[37] Katsanevakis S., Coll M., Piroddi C., Steenbeek J., Ben Rais Lasram F., Zenetos A., Cardoso A.C. (2014). Invading the Mediterranean Sea: Biodiversity patterns shaped by human activities. Front. Mar. Sci..

[38] Katsanevakis S., Poursanidis D., Hoffman R., Rizgalla J., Rothman S.B.-S., Levitt-Barmats Y., Hadjioannou L., Trkov D., Garmendia J.M., Rizzo M. (2020). Unpublished Mediterranean records of marine alien and cryptogenic species. BioInvasions Rec..

[39] Katsanevakis S., Olenin S., Puntila-Dodd R., Rilov G., Stæhr P.A.U., Teixeira H., Tsirintanis K., Birchenough S.N.R., Jakobsen H.H., Knudsen S.W. (2023). Marine invasive alien species in Europe: 9 years after the IAS Regulation. Front. Mar. Sci..

[40] Olenin S., Alemany F., Cardoso A.C., Gollasch S., Goulletquer P., Lehtiniemi M., McCollin T., Minchin D., Miossec L., Occhipinti-Ambrogi A. (2010). Marine Strategy Framework Directive—Task Group 2 Report. Non-indigenous species. EUR 24342 EN.

[41] UNEP/MAP (2016). State of the Mediterranean Marine Environment.

[42] UNEP/MAP (2017). Mediterranean Pollution Reduction and Prevention Report.

[43] UNEP/MAP (2021). Assessment of Marine and Coastal Environment in the Mediterranean.

[44] Katsanevakis S., Bogucarskis K., Gatto F., Vandekerkhove J., Deriu I., Cardoso A.C. (2012). Building the European Alien Species Information Network (EASIN): A novel approach for the exploration of distributed alien species data. BioInvasions Rec..

[45] Katsanevakis S., Deriu I., D’amico F., Nunes A.L., Sanchez S.P., Crocetta F., Arianoutsou M., Bazos I., Christopoulou A., Curto G. (2015). European Alien Species Information Network (EASIN): Supporting European policies and scientific research. Manag. Biol. Invasion.

[46] Costello M.J., Dekeyzer S., Galil B., Hutchings P., Katsanevakis S., Pagad S., Robinson T., Turon X., Vandepitte L., Vanhoorne B. (2021). Introducing the World Register of Introduced Marine Species (WRiMS). Manag. Biol. Invasions.

[47] Levin N., Bahar R., Çinar M.E., Daskalov G., Degiuseppe L., Folloni J., Goffredo S., Gomez S., Gosselin T., Karlsen K. (2014). Biodiversity data requirements for systematic conservation planning in the Mediterranean Sea. Mar. Ecol. Prog. Ser..

[48] Stulpinaite R., Hyams-Kaphzan O., Langer M.R. (2020). Alien and cryptogenic Foraminifera in the Mediterranean Sea: A revision of taxa as part of the EU 2020 Marine Strategy Framework Directive. Mediterr. Mar. Sci..

[49] Alve E. (1995). Benthic foraminiferal responses to estuarine pollution; a review. J. Foraminifer. Res..

[50] Vassallo P., Fabiano M., Vezzulli L., Sandulli R., Marques J., Jorgensen S. (2006). Assessing the health of coastal marine ecosystems: A holistic approach based on sediment micro and meio-benthic measures. Ecol. Indic..

[51] Schönfeld J., Alve E., Geslin E., Jorissen F., Korsun S., Spezzaferri S. (2012). The FOBIMO (FOraminiferal BIo-MOnitoring) initiative—Towards a standardised protocol for soft-bottom benthic foraminiferal monitoring studies. Mar. Micropaleontol..

[52] Jorissen F., Nardelli M.P., Almogi-Labin A., Barras C., Bergamin L., Bicchi E., El Kateb A., Ferraro L., McGann M., Morigi C. (2018). Developing Foram-AMBI for biomonitoring in the Mediterranean: Species assignments to ecological categories. Mar. Micropaleontol..

[53] Bouchet V.M., Frontalini F., Francescangeli F., Sauriau P.-G., Geslin E., Martins M.V.A., Almogi-Labin A., Avnaim-Katav S., Di Bella L., Cearreta A. (2021). Indicative value of benthic foraminifera for biomonitoring: Assignment to ecological groups of sensitivity to total organic carbon of species from European intertidal areas and transitional waters. Mar. Pollut. Bull..

[54] Parent B., Barras C., Bicchi E., Charrieau L.M., Choquel C., Bénéteau É., Maillet G.M., Jorissen F.J. (2021). Comparison of Four Foraminiferal Biotic Indices Assessing the Environmental Quality of Coastal Mediterranean Soft Bottoms. Water.

[55] O’Brien P.A., Polovodova-Asteman I., Bouchet V.M. (2021). Benthic foraminiferal indices and environmental quality assessment of transitional waters: A review of current challenges and future research perspectives. Water.

[56] Schmiedl G. (2019). Use of foraminifera in climate science. Oxford Research Encyclopedia of Climate Science.

[57] Chaabane S., de Garidel-Thoron T., Meilland J., Sulpis O., Chalk T.B., Brummer G.-J.A., Mortyn P.G., Giraud X., Howa H., Casajus N. (2024). Migrating is not enough for modern planktonic foraminifera in a changing ocean. Nature.

[58] Ying R., Monteiro F.M., Wilson J.D., Ödalen M., Schmidt D.N. (2024). Past foraminiferal acclimatization capacity is limited during future warming. Nature.

[59] Balsamo M., Semprucci F., Frontalini F., Coccioni R., Cruzado A. (2012). Meiofauna as a Tool for Marine Ecosystem Biomonitoring. Marine Ecosystems.

[60] Tietjen J.H. (1992). Abundance and biomass of metazoan meiobenthos in the deep sea. Deep-Sea Food Chains and the Global Carbon Cycle, Rowe, G.T., Pariente, V., Eds..

[61] Nomaki H., Ogawa N.O., Ohkouchi N., Suga H., Toyofuku T., Shimanaga M., Kitazato H. (2008). Benthic foraminifera as trophic links between phytodetritus and benthic metazoans: Carbon and nitrogen isotopic evidence. Mar. Ecol. Prog. Ser..

[62] Enge A.J., Nomaki H., Ogawa N.O., Witte U., Moeseneder M.M., Lavik G., Heinz P. (2011). Response of the benthic foraminiferal community to a simulated short-term phytodetritus pulse in the abyssal North Pacific. Mar. Ecol. Prog. Ser..

[63] Haynert K., Gluderer F., Pollierer M.M., Scheu S., Wehrmann A. (2020). Food spectrum and habitat-specific diets of benthic Foraminifera from the Wadden Sea–A fatty acid biomarker approach. Front. Mar. Sci..

[64] Altenbach A., Sarnthein M., Berger W.H., Smetacek V.S., Wefer G. (1989). Productivity Record in Benthic Foraminifera. Productivity of the Ocean: Present and Past.

[65] Gooday A.J., Levin L.A., Linke P., Heeger T. (1992). The Role of Benthic Foraminifera in Deep-Sea Food Webs and Carbon Cycling. Deep-Sea Food Chains and the Global Carbon Cycle, Rowe, G.T., Pariente, V., Eds..

[66] Gooday A.J., Nomaki H., Kitazato H. (2008). Modern deep-sea benthic foraminifera: A brief review of their morphology-based biodiversity and trophic diversity. Geol. Soc. Lond. Spec. Publ..

[67] van Lith Y., Langezaal A.M., de Nooijer L.J., van der Zwaan G.J. (2009). Benthic foraminiferal effect on nitrogen and carbon cycling. J. Foraminifer. Res..

[68] Glock N., Schönfeld J., Eisenhauer A., Hensen C., Mallon J., Sommer S. (2013). The role of benthic foraminifera in the benthic nitrogen cycle of the Peruvian oxygen minimum zone. Biogeosciences.

[69] Giovos I., Kleitou P., Poursanidis D., Poursanidis D., Batjakas I., Bernardi G., Crocetta F., Doumpas N., Kalogirou S., Kampouris T.E. (2019). Citizen-science for monitoring marine invasions and stimulating public engagement: A case project from the eastern Mediterranean. Biol. Invasions.

[70] Azzurro E., Sbragagli V., Cerri J., Bariche M., Bolognini L., Ben Souissi J., Berraho A., Bianchi C.N., Castriota L., Cioffi M. (2019). Climate Change, Biological Invasions, and the Shifting Distribution of Mediterranean Fishes: A Large-Scale Census Based on Local Ecological Knowledge. Glob. Change Biol..

[71] Langer M.R., Weinmann A.E., Lötters S., Rödder D. (2012). “Strangers” in paradise: Modelling the biogeographic range expansion of the foraminifera Amphistegina in the Mediterranean Sea. J. Foraminifer. Res..

[72] Guastella R., Marchini A., Caruso A., Cosentino C., Evans J., Weinmann A., Langer M., Mancin N. (2019). Hidden invaders conquer the Sicily Channel and knock on the door of the Western Mediterranean Sea. Estuar. Coast. Shelf Sci..

[73] Perzia P., Cillari T., Crociata G., Deidun A., Falautano M., Franzitta G., Galdies J., Maggio T., Vivona P., Castriota L. (2023). Using Local Ecological Knowledge to Search for Non-Native Species in Natura 2000 Sites in the Central Mediterranean Sea: An Approach to Identify New Arrivals and Hotspot Areas. Biology.

[74] Larsen A.R. (1976). Studies of Recent *Amphistegina*, Taxonomy and Some Ecological Aspects. Israel J. Earth Sci..

[75] Uchio T. (1962). Influence of the River Shinano on Foraminifera and Sediment Grain Size Distribution. Publ. Seto Mar. Biol. Lab..

[76] Caruso A., Cosentino C. (2014). The First Colonization of the Genus *Amphistegina* and Other Exotic Benthic Foraminifera of the Pelagian Islands and South-Eastern Sicily (Central Mediterranean Sea). Mar. Micropaleontol..

[77] Mouanga G.H., Langer M.R. (2014). At the Front of Expanding Ranges: Shifting Community Structures at Amphisteginid Species Range Margins in the Mediterranean Sea. Neues Jahrb. Geol. Paläontol. Abh..

[78] Bouchet V.M.P., Pavard J.-C., Holzmann M., McGann M., Armynot du Châtelet E., Courleux A., Pezy J.-P., Dauvin J.-C., Seuront L. (2023). The Invasive Asian Benthic Foraminifera *Trochammina hadai* Uchio, 1962: Identification of a New Local in Normandy (France) and a Discussion on Its Putative Introduction Pathways. Aquat. Invasions.

[79] Guastella R., Evans J., Mancin N., Caruso A., Marchini A. (2023). Assessing the Effect of *Amphistegina lobifera* Invasion on Infralittoral Benthic Foraminiferal Assemblages in the Sicily Channel (Central Mediterranean). Mar. Environ. Res..

[80] Weinmann A.E., Koukousioura O., Triantaphyllou M.V., Langer M.R. (2023). Invasive Shallow-Water Foraminifera Impacts Local Biodiversity Mostly at Densities Above 20%: The Case of Corfu Island. Web Ecol..

[81] Cosentino C., Guastella R., Mancin N., Caruso A. (2024). Spatial and Vertical Distribution of the Genus *Amphistegina* and Its Relationship with the Indigenous Benthic Foraminiferal Assemblages in the Pelagian Archipelago (Central Mediterranean Sea). Mar. Micropaleontol..

[82] Rocha L.A., Aleixo A., Allen G., Almeda F., Baldwin C.C., Barclay M.V., Bates J.M., Bauer A.M., Benzoni F., Berns C.M. (2014). Specimen Collection: An Essential Tool. Science.

[83] Encarnação J., Teodósio M.A., Morais P. (2021). Citizen Science and Biological Invasions: A Review. Front. Environ. Sci..

[84] Grindell D.S., Collen J.D. (1976). *Virgulinella fragilis* n. sp. (Foraminiferida) from Wellington Harbour, New Zealand. Rev. Esp. Micropaleontol..

[85] Capotondi L., Mancin N., Cesari V., Dinelli E., Ravaioli M., Riminucci F. (2019). Recent Agglutinated Foraminifera from the North Adriatic Sea: What the Agglutinated Tests Can Tell. Mar. Micropaleontol..

[86] Pugnetti A., Bastianini M., Cataletto B., Grilli F., Ravaioli M., Bernardi Aubry F., Acri F., Camatti E., Pansera M., Finotto S., Capotondi L., Ravaioli M., Acosta A., Chiarini F., Lami A., Stanisci A., Tarozzi L., Mazzocchi M.G. (2021). IT12-M Alto Adriatico. La Rete Italiana per la Ricerca Ecologica di Lungo Termine. Lo Studio della Biodiversità e dei Cambiamenti.

[87] Tesi T., Miserocchi S., Goñi M.A., Turchetto M., Langone L., De Lazzari A., Albertazzi S., Correggiari A. (2011). Influence of Distributary Channels on Sediment and Organic Matter Supply in Event-Dominated Coastal Margins: The Po Prodelta as a Study Case. Biogeosciences.

[88] Riminucci F., Funari V., Ravaioli M., Capotondi L. (2022). Trace Metals Accumulation on Modern Sediments from Po River Prodelta, North Adriatic Sea. Mar. Pollut. Bull..

[89] Bernhard J.M. (2003). Potential Symbionts in Bathyal Foraminifera. Mar. Micropaleontol..

[90] Takata H., Seto K., Sakai S., Tanaka S., Takayasu K. (2005). Correlation of *Virgulinella fragilis* Grindell & Collen (Benthic Foraminiferid) with Near-Anoxia in Aso-kai Lagoon, Central Japan. J. Micropalaeontol..

[91] Erbacher J., Nelskamp S. (2006). Comparison of Benthic Foraminifera Inside and Outside a Sulphur-Oxidizing Bacterial Mat from the Present Oxygen-Minimum Zone off Pakistan (NE Arabian Sea). Geophys. Res. Abst..

[92] Glock N. (2023). Benthic Foraminifera and Gromiids from Oxygen-Depleted Environments–Survival Strategies, Biogeochemistry, and Trophic Interactions. Biogeosciences.

[93] Tsuchiya M., Toyofuku T., Uematsu K., Brüchert V., Collen J., Yamamoto H., Kitazato H. (2015). Cytologic and Genetic Characteristics of Endobiotic Bacteria and Kleptoplasts of *Virgulinella fragilis* (Foraminifera). J. Eukaryot. Microbiol..

[94] Tsuchiya M., Grimm G.W., Heinz P., Stögerer K., Ertan K.T., Collen J., Brüchert V., Hemleben C., Hemleben V., Kitazato H. (2009). Ribosomal DNA Shows Extremely Low Genetic Divergence in a World-Wide Distributed, but Disjunct and Highly Adapted Marine Protozoan (*Virgulinella fragilis*, Foraminiferida). Mar. Micropaleontol..

[95] Artegiani A., Paschini E., Russo A., Bregant D., Raicich F., Pinardi N. (1997). The Adriatic Sea General Circulation. Part I: Air–Sea Interactions and Water Mass Structure. J. Phys. Oceanogr..

[96] Poulain P.M., Kourafalou V.H., Cushman-Roisin B., Cushman-Roisin B., Gačić M., Poulain P.M., Artegiani A. (2001). Northern Adriatic Sea. Physical Oceanography of the Adriatic Sea.

[97] Grilli F., Accoroni S., Acri F., Bernardi Aubry F., Bergami C., Cabrini M., Campanelli A., Giani M., Guicciardi S., Marini M. (2020). Seasonal and Interannual Trends of Oceanographic Parameters over 40 Years in the Northern Adriatic Sea in Relation to Nutrient Loadings Using the EMODnet Chemistry Data Portal. Water.

[98] Trincardi F., Cattaneo A., Asioli A., Correggiari A., Langone L. (1996). Stratigraphy of the Late-Quaternary Deposits in the Central Adriatic Basin and the Record of Short-Term Climatic Events. Mem.-Ist. Ital. Di Idrobiologia.

[99] Cattaneo A., Correggiari A., Langone L., Trincardi F. (2003). The Late-Holocene Gargano Subaqueous Delta, Adriatic Shelf: Sediment Pathways and Supply Fluctuations. Mar. Geol..

[100] Storms J.E.A., Weltje J., Terra G.J., Cattaneo A., Trincardi F. (2008). Coastal Dynamics under Conditions of Rapid Sea-Level Rise: Late Pleistocene to Early Holocene Evolution of Barrier–Lagoon Systems on the Northern Adriatic Shelf (Italy). Quat. Sci. Rev..

[101] Lipizer M., Partescano E., Rabitti A., Giorgetti A., Crise A. (2014). Qualified Temperature, Salinity and Dissolved Oxygen Climatologies in a Changing Adriatic Sea. Ocean Sci..

[102] Wang X.H., Pinardi N. (2002). Modeling the Dynamics of Sediment Transport and Resuspension in the Northern Adriatic Sea. J. Geophys. Res. Space Phys..

[103] Davolio S., Stocchi P., Benetazzo A., Böhm E., Riminucci F., Ravaioli M., Li X.-M., Carniel S. (2015). Exceptional Bora Outbreak in Winter 2012: Validation and Analysis of High-Resolution Atmospheric Model Simulations in the Northern Adriatic Area. Dyn. Atmos. Ocean..

[104] Vilibić I., Zemunik P., Šepić J., Dunić N., Marzouk O., Mihanović H., Denamiel C., Precali R., Djakovac T. (2019). Present Climate Trends and Variability in Thermohaline Properties of the Northern Adriatic Shelf. Ocean Sci..

[105] Kourafalou V.H. (1999). Process Studies on the Po River Plume, North Adriatic Sea. J. Geophys. Res..

[106] Struglia M.V., Mariotti A., Filograsso A. (2004). River Discharge into the Mediterranean Sea: Climatology and Aspects of the Observed Variability. J. Clim..

[107] Ludwig W., Dumont E., Meybeck M., Heussner S. (2009). River Discharges of Water and Nutrients to the Mediterranean and Black Sea: Major Drivers for Ecosystem Changes During Past and Future Decades?. Prog. Oceanogr..

[108] Danovaro R., Boero F., Sheppard C. (2019). Italian Seas. World Seas: An Environmental Evaluation.

[109] Aragão L., Mentaschi L., Pinardi N., Verri G., Senatore A., Di Sabatino S. (2024). The Freshwater Discharge into the Adriatic Sea Revisited. Front. Clim..

[110] Cozzi S., Giani M. (2011). River water and nutrient discharges in the Northern Adriatic Sea: Current importance and long term changes. Cont. Shelf Res..

[111] Montanari A. (2012). Hydrology of the Po River: Looking for Changing Patterns in River Discharge. Hydrol. Earth Syst. Sci..

[112] Marini M., Grilli F. (2023). The Role of Nitrogen and Phosphorus in Eutrophication of the Northern Adriatic Sea: History and Future Scenarios. Appl. Sci..

[113] Coppola E., Verdecchia M., Giorgi F., Colaiuda V., Tomassetti B., Lombardi A. (2014). Changing Hydrological Conditions in the Po Basin under Global Warming. Sci. Total Environ..

[114] Vezzoli R., Mercogliano P., Pecora S., Zollo A., Cacciamani C. (2015). Hydrological Simulation of Po River (North Italy) Discharge under Climate Change Scenarios Using the RCM COSMO-CLM. Sci. Total Environ..

[115] Nelson C.A., Morgan J.P. (1970). Hydrography, Sediment Dispersal, and Recent Historical Development of the Po River Delta, Italy. Deltaic Sedimentation, Modern and Ancient.

[116] Frignani M., Langone L., Ravaioli M., Sorgente D., Alvisi F., Albertazzi S. (2005). Fine Sediment Mass Balance in the Western Adriatic Continental Shelf over a Century Time Scale. Mar. Geol..

[117] Brambati A., Ciabatti M., Fanzutti G.P., Marabini F., Marocco R. (1983). A New Sedimentological Textural Map of the Northern and Central Adriatic Sea. Boll. Oceanogr. Teor. Appl..

[118] Pellegrini C., Tesi T., Schieber J., Bohacs K.M., Rovere M., Asioli A., Nogarotto A., Trincardi F. (2021). Fate of terrigenous organic carbon in muddy clinothems on continental shelves revealed by stratal geometries: Insight from the Adriatic sedimentary archive. Glob. Planet. Change.

[119] Boldrin A., Langone L., Miserocchi S., Turchetto M., Acri F. (2005). Po River plume on the Adriatic continental shelf: Dispersion and sedimentation of dissolved and suspended matter during different river discharge rates. Mar. Geol..

[120] Van der Zwaan G.J., Jorissen F.J., Tyson R.V., Pearson T.H. (1991). Biofacial Patterns in River-Induced Shelf Anoxia. Modern and Ancient Continental Shelf Anoxia.

[121] Alvisi F., Giani M., Ravaioli M., Giordano P. (2013). Role of sedimentary environment in the development of hypoxia and anoxia in the NW Adriatic shelf (Italy). Estuar. Coast. Shelf Sci..

[122] Albertazzi S., Bopp R.F., Frignani M., Merlin O.H., Vitturi L.M., Ravaioli M., Simpson H.J., Tassi Pelati L., Triulzi C. (1984). Cs-137 as a Tracer for Processes of Marine Sedimentation in the Vicinity of the Po River Delta (Northern Adriatic Sea). Mem. Soc. Geol. Italy.

[123] Amorosi A., Sammartino I., Dinelli E., Campo B., Guercia T., Trincardi F., Pellegrini C. (2022). Provenance and sediment dispersal in the Po-Adriatic source-to-sink system unraveled by bulk-sediment geochemistry and its linkage to catchment geology. Earth-Sci. Rev..

[124] Tesi T., Langone L., Giani M., Ravaioli M., Miserocchi S. (2013). Source, Diagenesis, and Fluxes of Particulate Organic Carbon along the Western Adriatic Sea (Mediterranean Sea). Mar. Geol..

[125] Barra E., Riminucci F., Dinelli E., Albertazzi S., Giordano P., Ravaioli M., Capotondi L. (2020). Natural Versus Anthropic Influence on the North Adriatic Coast Detected by Geochemical Analyses. Appl. Sci..

[126] Furlan E., Torresan S., Critto A., Lovato T., Solidoro C., Lazzari P., Marcomini A. (2019). Cumulative Impact Index for the Adriatic Sea: Accounting for interactions among climate and anthropogenic pressures. Sci. Total Environ..

[127] Barbanti A., Sarretta A., Venier C., Depellegrin D., Bellacicco S., Farella G., Menegon S., Lorito S., Ghezzo M., Grati F., Barbanti A., Perini L. (2018). Fra la Terra e il Mare: Analisi e Proposte per la Pianificazione dello Spazio Marittimo in Emilia-Romagna.

[128] Giani M., Djakovac T., Degobbis D., Cozzi S., Solidoro C., Umani S.F. (2012). Recent changes in the marine ecosystems of the northern Adriatic Sea. Estuar. Coast. Shelf Sci..

[129] Gallina V., Torresan S., Zabeo A., Critto A., Glade T., Marcomini A. (2020). A Multi-Risk Methodology for the Assessment of Climate Change Impacts in Coastal Zones. Sustainability.

[130] Marchini A., Ferrario J., Sfriso A., Occhipinti-Ambrogi A. (2015). Current status and trends of biological invasions in the Lagoon of Venice, a hotspot of marine NIS introductions in the Mediterranean Sea. Biol. Invasions.

[131] Slišković M., Piria M., Nerlović V., Ivelja K.P., Gavrilović A., Mrčelić G.J. (2021). Non-indigenous species likely introduced by shipping into the Adriatic Sea. Mar. Policy.

[132] Russo E., Monti M.A., Mangano M.C., Raffaetà A., Sarà G., Silvestri C., Pranovi F. (2020). Temporal and spatial patterns of trawl fishing activities in the Adriatic Sea (Central Mediterranean Sea, GSA17). Ocean. Coast. Manag..

[133] Petranich E., Covelli S., Acquavita A., De Vittor C., Faganeli J., Contin M. (2018). Benthic nutrient cycling at the sediment-water interface in a lagoon fish farming system (northern Adriatic Sea, Italy). Sci. Total Environ..

[134] Giani M., Boldrin A., Matteucci G., Frascari F., Gismondi M., Rabitti S. (2001). Downward Fluxes of Particulate Carbon, Nitrogen, and Phosphorus in the North-Western Adriatic Sea. Sci. Total Environ..

[135] Frascari F., Spagnoli F., Marcaccio M., Giordano P. (2006). Anomalous Po River Flood Event Effects on Sediments and the Water Column of the Northwestern Adriatic Sea. Clim. Res..

[136] Guarnieri A., Pinardi N., Oddo P., Bortoluzzi G., Ravaioli M. (2013). Impact of Tides in a Baroclinic Circulation Model of the Adriatic Sea. J. Geophys. Res. Ocean..

[137] Braga F., Zaggia L., Bellafiore D., Bresciani M., Giardino C., Lorenzetti G., Maicu F., Manzo C., Riminucci F., Ravaioli M. (2017). Mapping Turbidity Patterns in the Po River Prodelta Using Multi-Temporal Landsat 8 Imagery. Estuar. Coast. Shelf Sci..

[138] Frascari F., Frignani M., Giordani P., Guerzoni S., Ravaioli M. (1984). Sedimentological and Geochemical Behavior of Heavy Metals in the Area Near the Po River Delta. Mem. Soc. Geol. Italy.

[139] Frignani M., Langone L. (1991). Accumulation Rates and 137Cs Distribution in Sediments off the Po River Delta and the Emilia-Romagna Coast (Northwestern Adriatic Sea, Italy). Cont. Shelf Res..

[140] Matteucci G., Frascari F. (1997). Fluxes of Suspended Materials in the North Adriatic Sea (Po Prodelta Area). Water Air Soil Pollut..

[141] Ianni C., Magi E., Rivaro P., Ruggieri N. (2000). Trace Metals in Adriatic Coastal Sediments: Distribution and Speciation Pattern. Toxic. Environ. Chem..

[142] Alvisi F. (2009). A Simplified Approach to Evaluate Sedimentary Organic Matter Fluxes and Accumulation on the NW Adriatic Shelf (Italy). Chem. Ecol..

[143] Spagnoli F., Dinelli E., Giordano P., Marcaccio M., Zaffagnini F., Frascari F. (2014). Sedimentological, Biogeochemical and Mineralogical Facies of Northern and Central Western Adriatic Sea. J. Mar. Syst..

[144] Alvisi F., Cibic T., Fazi S., Bongiorni L., Relitti F., Del Negro P. (2019). Role of Depositional Dynamics and Riverine Input in Shaping Microbial Benthic Community Structure of Po Prodelta System (NW Adriatic, Italy). Estuar. Coast. Shelf Sci..

[145] D’Onofrio R., Capotondi L. North Adriatic Foraminifera Collection (NAdFC). Version 1.2. Consiglio Nazionale delle Ricerche, Istituto di Scienze Marine di Venezia. Occurrence Dataset. https://cloud.gbif.org/eca/resource?r=nadfc_foraminifera&v=1.2.

[146] Böhm E., Riminucci F., Bortoluzzi G., Colella S., Acri F., Santoleri R., Ravaioli M. (2016). Operational Use of Continuous Surface Fluorescence Measurements Offshore Rimini to Validate Satellite-Derived Chlorophyll Observations. J. Oper. Oceanogr..

[147] Ravaioli M., Bergami C., Riminucci F., Langone L., Cardin V., Di Sarra A., Aracri A., Bastianini M., Bensi M., Bergamasco A. (2016). The RITMARE Italian Fixed-Point Observatory Network (IFON) for Marine Environmental Monitoring: A Case Study. J. Oper. Oceanogr..

[148] Sherwood C.R., Carniel S., Cavaleri L., Chiggiato J., Himangshu D., Doyle J., Harris C., Niedoroda A., Pullen J., Reed C. (2004). Sediment Dynamics in the Adriatic Sea Investigated with Coupled Models. Oceanography.

[149] Harris C.K., Sherwood C.R., Signell R.P., Bever A.J., Warner J.C. (2008). Sediment Dispersal in the Northwestern Adriatic Sea. J. Geophys. Res. Oceans.

[150] Boldrin A., Carniel S., Giani M., Marini M., Bernardi Aubry F., Campanelli A., Grilli F., Russo A. (2009). Effects of Bora Wind on Physical and Biogeochemical Properties of Stratified Waters in the Northern Adriatic. J. Geophys. Res. Atmos..

[151] Foglini F., Bosman A., Correggiari A., Remia A., Madricardo F., Prampolini M., Fontolan G., Biscotti E., Ferrero S., Pizzeghello N. (2020). Carta Batimorfologica dell’Adriatico Settentrionale.

[152] Bastianini M., Riminucci F., Capondi L., Barra E., Pasqual S., Casotti R., Trano A.C., Van Dijk M., Mauro C., Fabbro C. (2017). Rapporto sulle attività oceanografiche, biologiche, geologiche e di manutenzione della stazione meda S1-GB svolte durante la campagna oceanografica LTER-ANOC16 (26-30 aprile 2016) con N/O Dallaporta nel Mare Adriatico settentrionale. Rapporto Tecnico CNR-ISMAR Bologna.

[153] Bastianini M., Riminucci F., Pansera M., Coluccelli A., Casotti R., Dal Passo E., Dametto L., Van Dijk M., Russo E., Titocci J. (2017). Rapporto sulle attività biologiche, oceanografiche, geologiche e di manutenzione della stazione Boa E1 svolte durante la campagna INTERNOS17 (14-21 marzo 2017) con N/O Minerva Uno nel Mare Adriatico centro-settentrionale. Rapporto Tecnico CNR-ISMAR Bologna.

[154] Bastianini M., Riminucci F., Bernardi Aubry F., Casotti R., Coluccelli A., Trano A.C., Epinoux A., Donnarumma V. (2019). Rapporto sulle attività biologiche, oceanografiche, geologiche svolte durante la campagna INTERNOS19 (20-28 Febbraio 2019) con N/O Dallaporta nel mare Adriatico centro-settentrionale. Rapporto Tecnico CNR-ISMAR.

[155] Schröder C.J., Scott D.B., Medioli F.S., Bernstein B.B., Hessler R.R. (1988). Larger Agglutinated Foraminifera: Comparison of Assemblages from Central North Pacific and Western North Atlantic (Nares Abyssal Plain). J. Foraminifer. Res..

[156] Duchemin G., Fontanier C., Jorissen F.J., Barras C., Griveaud C. (2007). Living Small-Sized (63–150 μm) Foraminifera from Mid-Shelf to Mid-Slope Environments in the Bay of Biscay. J. Foraminifer. Res..

[157] Loeblich A.J.R., Tappan H. (1987). Foraminiferal Genera and Their Classification, 1–2.

[158] Barmawidjaja D.M., Jorissen F.J., Puskaric S., Van der Zwaan G.J. (1992). Microhabitat Selection by Benthic Foraminifera in the Northern Adriatic Sea. J. Foraminifer. Res..

[159] Cimerman F., Langer M.R. (1991). Mediterranean Foraminifera.

[160] Jorissen F.J. (1987). The Distribution of Benthic Foraminifera in the Adriatic Sea. Mar. Micropaleontol..

[161] Jorissen F.J. (1988). Benthic Foraminifera from the Adriatic Sea: Principles of Phenotypic Variation.

[162] Hayward B.W., Cedhagen T., Kaminski M., Gross O. (2011). World Modern Foraminifera Database. http://www.marinespecies.org/foraminifera/index.php.

[163] Phillips S.J., Dudík M. (2008). Modeling of Species Distributions with Maxent: New Extensions and a Comprehensive Evaluation. Ecography.

[164] Elith J., Phillips S.J., Hastie T., Dudík M., Chee Y.E., Yates C.J. (2010). A Statistical Explanation of MaxEnt for Ecologists. Divers. Distrib..

[165] Merow C., Smith M.J., Silander J.A. (2013). A Practical Guide to MaxEnt for Modeling Species’ Distributions: What It Does, and Why Inputs and Settings Matter. Ecography.

[166] Delliou A.V., Antoniadou C., Chintiroglou C.C. Diversity of Benthic Foraminifera from Sublittoral Sediments in Thermaikos Gulf (North Aegean Sea) with New Mediterranean Records. Proceedings of the 43rd CIESM Congress.

[167] Jorissen F., Fouet M., Armynot du Châtelet É., Barras C., Bouchet V., Daviray M., Francescangeli F., Geslin E., Le Moigne D., Licari L. (2023). Foraminifères Estuariens de la Façade Atlantique Française: Guide de Détermination.

[168] Hayward B.W., Grenfell H.R., Reid C.M., Hayward K.A. (1999). Recent New Zealand Shallow-Water Benthic Foraminifera: Taxonomy, Ecologic Distribution, Biogeography, and Use in Paleoenvironmental Assessments.

[169] Apthorpe M. (1980). Foraminiferal distribution in the estuarine Gippsland Lakes System, Victoria. Proc. Royal. Soc. Victoria.

[170] McCulloch I. (1977). Qualitative Observations on Recent Foraminiferal Tests with Emphasis on the Eastern Pacific.

[171] Cardich J., Gutiérrez D., Romero D., Pérez A., Quipúzcoa L., Marquina R., Rathburn A. (2015). Calcareous benthic foraminifera from the upper central Peruvian margin: Control of the assemblage by pore water redox and sedimentary organic matter. Mar. Ecol. Prog. Ser..

[172] Altenbach A.V., Struck U., Graml M., Emeis K. The genus *Virgulinella* in oxygen deficient, oligotrophic, or polluted sediments. Proceedings of the Forams 2002 International Symposium on Foraminifera.

[173] Ertan K.T., Hemleben V., Hemleben C. (2004). Molecular evolution of some selected benthic foraminifera as inferred from sequences of the small subunit ribosomal DNA. Mar. Micropaleontol..

[174] Leiter C., Altenbach A.V. (2010). Benthic foraminifera from the diatomaceous mud belt off Namibia: Characteristic species for severe anoxia. Palaeontol. Electron..

[175] Bermudez P.G., Seiglie G.A. (1963). Estudio sistematico de los foraminiferos del Gulfo Cariaco. Bol. Inst. Oceanogr. Univ. Oriente.

[176] Sellier de Civrieux J.M. (1977). Foraminiferos indicadores de comunidades bentonicas recientes en Venezuela. Parte II. Ecologia y distribucion de los foraminiferos mas frecuentes de la plataforma continental en el Parque Nacional Mochima. Bol. Inst. Oceanogr. Univ. Oriente.

[177] Bhatia S.B., Kumar S. (1976). Recent benthonic foraminifera from the inner shelf area around Anjidiv Island, off Binge, west coast of India. Ecolo. Biol. Marit. Sediments Spec. Publ..

[178] Saravanan P., Gupta A.K., Zheng H., Panigrahi M.K., Tiwari S.K., Rai S.K., Prakasam M. (2020). Response of shallow-sea benthic foraminifera to environmental changes off the coast of Goa, eastern Arabian Sea, during the last ~6100 cal yr BP. Geol. Mag..

[179] Culver S.J., Buzas M.A. (1981). Distribution of recent benthic foraminifera in the Gulf of Mexico. Smithson. Contrib. Mar. Sci..

[180] Revets S.A. (1991). The nature of *Virgulinella* Cushman, 1932, and the implications for its classification. J. Foraminifer. Res..

[181] Salonen I.S., Chronopoulou P.M., Bird C., Reichart G.J., Koho K.A. (2019). Enrichment of intracellular sulphur cycle–associated bacteria in intertidal benthic foraminifera revealed by 16S and aprA gene analysis. Sci. Rep..

[182] Dukan N., Cornelis I., Brosens D., Derycke S. Vertical and Horizontal Environmental DNA (eDNA) Patterns of Fish in a Shallow and Well-Mixed North Sea Area. Version 1.7. Flanders Research Institute for Agriculture, Fisheries and Food (ILVO). Occurrence Dataset. https://ipt.inbo.be/resource?r=ilvo-metabarcoding-1&v=1.7.

[183] Pavard Richirt J., Bouchet V.M.P., Holzmann M., Mcgann M., Du Châtelet E.A., Pezy J.P., Dauvin J.C., Seuront L. Unexpected high records of non-indigenous foraminiferal species in the eastern English Channel. Proceedings of the FORAMS 2023.

[184] Fontanier C., Dissard D., Ruffine L., Mamo B., Ponzevera E., Pelleter E., Savignac F. (2018). Living (stained) deep-sea foraminifera from the Sea of Marmara: A preliminary study. Deep Sea Res. Part II Topical Stud. Oceanogr..

[185] Albano P.G., Sabbatini A., Lattanzio J., Päßler J.F., Steger J., Hua Q., Kaufman D.S., Szidat S., Zuschin M., Negri A. (2022). Alleged Lessepsian foraminifera prove native and suggest Pleistocene range expansions into the Mediterranean Sea. Mar. Ecol. Prog. Ser..

[186] Reuss A.E. (1861). Beiträge zur Kenntniss der tertiären Foraminiferen-Fauna. I. Die Foraminiferen des Crag’s von Antwerpen. II. Die Foraminiferen von Dingden in Westphalen. Sitzungsberichte der Kaiserlichen Akademie der Wissenschaften. Math.-Naturwissenschaftliche Cl..

[187] Todd R., Brönnimann P. (1957). Recent foraminifera and thecamoebina from the eastern Gulf of Paria. Cushman Found. Foraminifer. Res. (Spec. Publ.).

[188] Loeblich A.R., Tappan H., Moore R.C. (1964). Sarcodina (chiefly “Thecamoebians” and Foraminiferida). Treatise on Invertebrate Paleontology-(c) Protista 2 (2).

[189] Haman D. (1977). Comments on the genus *Virgulinella* Cushman, 1932 (Foraminiferida). Tulane Stud. Geol. Paleontol..

[190] Stolyarov A.S. (2001). Upper Oligocene Virgulinella Bed of the Ciscaucasus, Volga–Don, and Mangyshlak Regions (Central Part of the Eastern Paratethys). Lithol. Miner. Resour..

[191] Amorosi A., Barbieri G., Bruno L., Campo B., Drexler T.M., Hong W., Rossi V., Sammartino I., Scarponi D., Vaiani S.C. (2019). Three-fold nature of coastal progradation during the Holocene eustatic highstand, Po Plain, Italy–close correspondence of stratal character with distribution patterns. Sedimentology.

[192] Langer M.R., Hottinger L. (2000). Biogeography of selected “larger” foraminifera. Micropaleontology.

[193] Pawlowski J., Lejzerowicz F., Esling P. (2014). Next-generation environmental diversity surveys of foraminifera: Preparing the future. Biol. Bull..

[194] Holzmann M., Nguyen N.L., Angeles I.B., Pawlowski J. (2024). BFR2: A curated ribosomal reference dataset for benthic foraminifera. Sci. Data.

[195] Alve E., Goldstein S.T. (2003). Propagule transport as a key method of dispersal in benthic foraminifera (Protista). Limnol. Oceanogr..

[196] Alve E. (1999). Colonization of new habitats by benthic foraminifera: A review. Earth Sci. Rev..

[197] Alve E., Goldstein S.T. (2002). Resting stage in benthic foraminiferal propagules: A key feature for dispersal? Evidence from two shallow-water species. J. Micropal..

[198] Moodley L., van der Zwaan G.J., Herman P.M.J., Kempers L., Breugel P. (1997). van. Differential response of benthic meiofauna to anoxia with special reference to Foraminifera (Protista: Sarcodina). Mar. Ecol. Prog. Ser..

[199] Guy-Haim T., Hyams-Kaphzan O., Yeruham E., Almogi-Labin A., Carlton J.T. (2017). A novel marine bioinvasion vector: Ichthyochory, live passage through fish. Limnol. Oceanogr. Lett..

[200] Servello G., Andaloro F., Azzurro E., Castriota L., Catra M., Chiarore A., Crocetta F., D’alessandro M., Denitto F., Froglia C. (2019). Marine alien species in Italy: A contribution to the implementation of descriptor D2 of the marine strategy framework directive. Mediterr. Mar. Sci..

[201] Molnar J.L., Gamboa R.L., Revenga C., Spalding M.D. (2008). Assessing the global threat of invasive species to marine biodiversity. Front. Ecol. Environ..

[202] Cerrano C., Cebrian D., Requena Moreno S., Bakran-Petricioli T., Bastari A., Fraschetti S., Huete-Stauffer C., Ferretti F., Micheli F., Ponti M. (2015). Description of the ecology and identification of the areas that may deserve to be protected. *United Nations Environment Programme/Mediterranean Action Plan (UNEP/MAP) Regional Activity Centre for Specially Protected Areas (RAC/SPA)*. https://www.unep.org/.

[203] Novikov M., Pakhomova S., Berezina A., Yakushev E. (2024). Model-Based Analysis of the Oxygen Budget in the Black Sea Water Column. Water.

[204] Capet A., Stanev E.V., Beckers J.-M., Murray J.W., Grégoire M. (2016). Decline of the Black Sea oxygen inventory. Biogeosciences.

[205] Goulletquer P., Bachelet G., Sauriau P.-G., Noël P., Leppäkoski E., Gollasch S., Olenin S. (2002). Open Atlantic coast of Europe—A century of introduced species into French waters. Invasive Aquatic Species of Europe. Distribution, Impacts and Management.

[206] Occhipinti-Ambrogi A., Marchini A., Cantone G., Castelli A., Chimenz C., Cormaci M., Froglia C., Furnari G., Gambi M.C., Giaccone G. (2011). Alien species along the Italian coasts: An overview. Biol. Invasions.

[207] Zenetos A., Çinar M.E., Crocetta F., Golani D., Rosso A., Servello G., Shenkar N., Turon X., Verlaque M. (2017). Uncertainties and validation of alien species catalogues: The Mediterranean as an example. Estuar. Coast. Shelf Sci..

[208] Mosbahi N., Pezy J.-P., Neifar L., Dauvin J.-C. (2021). Ecological status assessment and non-indigenous species in industrial and fishing harbours of the Gulf of Gabès (central Mediterranean Sea). Environ. Sci. Pollut. Res..

[209] UNCTAD/RMT/2019/Corr.1 United Nations Conference on Trade and Development Sales No. E.19.II.D.20 January 2020. https://unctad.org/system/files/official-document/rmt2019_en.pdf.

[210] UNCTAD/RMT/2024/Corr.1 United Nations Conference on Trade and Development Sales No. E.24.II.D.24 October 2024. https://unctad.org/system/files/official-document/rmt2024_en.pdf.

[211] UNEP/MAP State of the Mediterranean Marine and Coastal Environment; UNEP/MAP—Barcelona Convention 2012. www.unepmap.org/unepmap/what-we-do/monitoring-and-assessments.

[212] Di Blasio L., Chiesa S., Arcangeli G., Donadelli V., Marino G. (2023). Alien Species Associated with New Introductions and Translocations of Commercial Bivalves in Italian Marine Waters. Sustainability.

[213] Robert R., Sánchez J.L., Pérez-Parallé L., Ponis E., Kamermans P., O’mahoney M. (2013). A glimpse on the mollusc industry in Europe. Aquac. Eur..

[214] Martínez J., García-Ladona E., Ballabrera-Poy J., Isern-Fontanet J., González-Motos S., Allegue J.M., González-Haro C. (2024). Atlas of surface currents in the Mediterranean and Canary–Iberian–Biscay waters. J. Oper. Oceanogr..

[215] Rožič P.Ž., Vidović J., Ćosović V., Hlebec A., Rožič B., Dolenec M. (2022). Multiparametric Approach to Unravelling the Geoenvironmental Conditions in Sediments of Bay of Koper (NE Adriatic Sea): Indicators of Benthic Foraminifera and Geochemistry. Front. Mar. Sci..

[216] Ćosović V., Gajski N., Ptčiek A., Vidović J., Kružić P. (2016). The distribution of benthic foraminifera in Cladocora caespitosa coral banks of the Veliko Jezero sediments (Mljet National Park, eastern Adriatic Sea). Neues Jahrb. Geol. Paläontologie-Abh..

[217] Franzo A., Caffau M., Nasi F., Marrocchino E., Paletta M.G., Bazzaro M., Cibic T. (2023). Benthic foraminifera for the ecological status assessment of tourist marinas. Ecol. Indic..

[218] Rostami M.A., Frontalini F., Armynot du Châtelet E., Francescangeli F., Alves Martins M.V., De Marco R., Dinelli E., Tramontana M., Dyer L.A., Abraham R. (2023). Understanding the Distributions of Benthic Foraminifera in the Adriatic Sea with Gradient Forest and Structural Equation Models. Appl. Sci..

[219] Resig S.M. (1974). Recent foraminifera from a landlocked Hawaiian lake. J. Foraminifer. Res..

[220] Androulidakis Y., Makris C., Kombiadou K., Krestenitis Y., Stefanidou N., Antoniadou C., Krasakopoulou E., Kalatzi M.-I., Baltikas V., Moustaka-Gouni M. (2024). Oceanographic Research in the Thermaikos Gulf: A Review over Five Decades. J. Mar. Sci. Eng..

[221] Gaglio M., Lanzoni M., Muresan A.N., Schirpke U., Castaldelli G. (2024). Quantifying intangible values of wetlands as instrument for conservation in the Po delta park (Italy). J. Environ. Manag..

[222] Brown C.J., Mendelsohn J.M., Thomson N., Boorman M. (2017). Checklist and analysis of the birds of Namibia as at 31 January 2016. Biodivers. Obs..

[223] Davis F., Szopa-Comley A., Rouse S., Caromel A., Arnell A., Basrur S., Bhola N., Brooks H., Costa-Domingo G., Cunningham C. (2024). State of the World’s Migratory Species.

[224] Wearne K., Underhill L.G. (2005). Walvis Bay, Namibia: A key wetland for waders and other coastal birds in southern Africa. Wader Study Group Bull..

[225] Bernhard J.M. (1986). Characteristic assemblages and morphologies of benthic foraminifera from anoxic, organic-rich deposits; Jurassic through Holocene. J. Foraminifer. Res..

[226] Bernhard J.M., Sen Gupta B.K. (1999). Foraminifera of oxygen-depleted environments. Mod. Foraminifera.

[227] Bernhard J.M., Sen Gupta B.K., Borne P.F. (1997). Benthic foraminiferal proxy to estimate dysoxic bottomwater oxygen concentrations; Santa Barbara Basin, U.S. Pacific continental margin. J. Foraminifer. Res..

[228] Kaiho K. (1994). Benthic foraminiferal dissolved-oxygen index and dissolved-oxygen levels in the modern ocean. Geology.

[229] Berthold W.U. (1976). Ultrastructure and function of wall perforations in *Patellina corrugata*, Williamson, foraminifera. J. Foraminifer. Res..

[230] Glock N., Eisenhauer A., Milker Y., Liebetrau V., Schonfeld J., Mallon J., Sommer S., Hensen C. (2011). Environmental influences on the pore density of *Bolivina spissa* (Cushman). J. Foraminifer. Res..

[231] Leutenegger S., Hansen H.J. (1979). Ultrastructural and radiotracer studies of pore function in foraminifera. Mar. Biol..

[232] Pérez-Cruz L., Machain Castillo M.L. (1990). Benthic foraminifera of the oxygen minimum zone, continental shelf of the Gulf of Tehuantepec, Mexico. J. Foraminifer. Res..

[233] Wang F., Yang S., Zhai B., Gong S., Wang J., Fu X., Ning Z. (2023). Pore density of the benthic foraminiferal test responded to the hypoxia off the Changjiang estuary in the East China Sea. Front. Mar. Sci..

[234] Moodley L., Hess C. (1992). Tolerance of infaunal benthic foraminifera for low and high oxygen concentrations. Biol. Bull..

[235] Johannesson K.J., Martin R.E. (1955). The bacteriological survey of Wellington Harbour—II. NZ J. Sci. Technol. B.

[236] Johannesson K.J., Martin R.E. (1955). The bacteriological survey of Wellington Harbour—III. NZ J. Sci. Technol. B.

[237] Murray J.W. (1991). Ecology and Palaeoecology of Benthic Foraminifera.

[238] Alvisi F., Cozzi S. (2016). Seasonal dynamics and long-term trend of hypoxia in the coastal zone of Emilia Romagna (NW Adriatic Sea, Italy). Sci. Total Environ..

[239] Giani M., Berto D., Rampazzo F., Savelli F., Alvisi F., Giordano P., Ravaioli M., Frascari F. (2009). Origin of sedimentary organic matter in the north-western Adriatic Sea. Estuar. Coast. Shelf Sci..

[240] Gross O. (2000). Influence of temperature, oxygen and food availability on the migrational activity of bathyal benthic foraminifera: Evidence by microcosm experiments. Hydrobiologia.

[241] Ernst S., Duijnstee I., Fontanier C., Jorissen F., van der Zwaan B. (2008). A comparison of foraminiferal infaunal distributions in field and experimental samples from 550-m depth in the Bay of Biscay, Deep Sea Res. Oceanogr. Res. Pap..

[242] Wollenburg J.E., Zittier Z.M.C., Bijma J. (2018). Insights into deep-sea life—Cibicidoides pachyderma substrate and pH-dependent behaviour following disturbance. Deep Sea Res. Oceanogr. Res. Pap..

[243] Jorissen F.J., de Stigter H.C., Widmark J.G.V. (1995). A conceptual model explaining benthic foraminiferal microhabitats. Mar. Micropaleontol..

[244] Gooday A.J., Rathburn A.E. (1999). Temporal variability in living deep-sea benthic foraminifera: A review. Earth Sci. Rev..

[245] Fontanier C., Jorissen F.J., Licari L., Alexandre A., Anschutz P., Carbonel P. (2002). Live benthic foraminiferal faunas from the Bay of Biscay: Faunal density, composition, and microhabitats. Deep. Sea Res. Part I Oceanogr. Res. Pap..

[246] Hoogakker B.A.A., Davis C., Wang Y., Kusch S., Nilsson-Kerr K., Hardisty D.S., Jacobel A., Reyes Macaya D., Glock N., Ni S. (2025). Reviews and syntheses: Review of proxies for low-oxygen paleoceanographic reconstructions. Biogeosciences.

[247] Widdel F. (1987). New types of acetate-oxidizing, sulfate-reducing *Desulfobacter* species, *D. hydrogenophilus* sp. nov., *D. latus* sp. nov., and *D. curvatus* sp. nov. Arch. Microbiol..

[248] Woehle C., Roy A.S., Glock N., Michels J., Wein T., Weissenbach J., Dagan T. (2022). Denitrification in foraminifera has an ancient origin and is complemented by associated bacteria. Proc. Natl. Acad. Sci. USA.

[249] Malagó A., Bouraoui F., Grizzetti B., De Roo A. (2019). Modelling nutrient fluxes into the Mediterranean Sea. J. Hydrol. Reg..

[250] Goineau A., Fontanier C., Jorissen F.J., Lansard B., Buscail R., Mouret A., Rabouille C. (2011). Live (stained) benthic foraminifera from the Rhône prodelta (Gulf of Lion, NW Mediterranean): Environmental controls on a river-dominated shelf. J. Sea Res..

[251] Badr-ElDin A.M., Charle C.M., El-Sabrouti M.A. (2019). Response of benthic foraminifera to coastal protection of the western coast of Alexandria, Egypt. Egypt. J. Aquat. Res..

[252] Takata H., Tanaka S., Seto K., Sakai S., Takayasu K., Khim B.K. (2014). Biotic response of benthic foraminifera in Aso-kai lagoon, central Japan, to changes in terrestrial climate and ocean conditions (~AD 700–1600). J. Paleolimnol..

